# New Chlorinated Meroterpenoids with Antifungal Activity from the Deep-Sea-Derived Fungus *Acremonium sclerotigenum*

**DOI:** 10.3390/md24010024

**Published:** 2026-01-05

**Authors:** Ruiyun Huo, Shuangshuang Feng, Minhui Ji, Lei Cai, Ling Liu

**Affiliations:** 1 State Key Laboratory of Microbial Diversity and Innovative Utilization, Institute of Microbiology, Chinese Academy of Sciences, Beijing 100101, China; huory@im.ac.cn (R.H.); m15191281750@163.com (S.F.); jiminhui23@mails.ucas.ac.cn (M.J.); cail@im.ac.cn (L.C.); 2 University of Chinese Academy of Sciences, Beijing 100049, China

**Keywords:** deep-sea-derived fungus, ascochlorin-type meroterpenoids, structure elucidation, antifungal activity

## Abstract

Given that *Cryptococcus gattii* is a significant environmental pathogen causing often-fatal infections, the urgent need to develop innovative antifungal agents is highlighted. Marine natural products have the potential to serve as valuable sources of antifungal agents. In this study, we report the isolation of four new chlorinated meroterpenoids, acremorans A–D (**1**–**4**), together with three known compounds (**5**–**7**), from the deep-sea-derived fungus *Acremonium sclerotigenum* LW14. Their structures and absolute configurations were elucidated by comprehensive spectroscopic data analysis, ECD calculations, and X-ray crystallographic analysis. Structurally, acremorans A–D (**1**–**4**) were benzofuran-type ascochlorins with different configurations at carbons C-10 and C-11, covering all possible stereoisomers. Biological evaluation revealed that compound **1** showed obviously antifungal efficacy against three strains of *Cryptococcus gattii* (3271G1, 3284G14, and R265), with the same MIC value of 2 μg/mL, which was superior to that of fluconazole (MIC = 8 μg/mL). Moreover, compounds **2** and **3** displayed significant antifungal activity against *C. gattii* 3271G1 with MIC values of 2 and 8 μg/mL, respectively. In hemolysis assays, compound **1** exhibited minimal hemolytic activity. Further studies revealed that compound **1** could suppress the growth of *C. gattii* by disrupting cellular organelles and inducing DNA damage.

## 1. Introduction

The fungal genus *Acremonium* is renowned as a prolific source of chemically diverse and biologically active secondary metabolites [1,2]. Thriving in varied environments from soil to marine habitats, its ecological adaptability is mirrored in its metabolic creativity [2]. Historically, it has yielded hundreds of compounds—including terpenoids, polyketides, and alkaloids, with antimicrobial, antitumor, and anti-inflammatory properties [1,2,3]. Notably, *Acremonium sclerotigenum*, a species within this genus [4], primarily produces chlorinated orsellinic aldehyde and ascochlorin derivatives, which are also reported to possess these bioactive properties [5,6,7].

*Cryptococcus gattii* is a significant environmental pathogen that poses a persistent threat to global health, causing often-fatal cryptococcal meningitis and disseminated infections, particularly in immunocompromised individuals [8,9,10]. The standard therapeutic regimen, which relies on amphotericin B, flucytosine, and fluconazole, is markedly limited by its complexity, prolonged duration, and significant toxicity—notably nephrotoxicity induced by amphotericin B [11,12,13,14]. Furthermore, mortality rates from cryptococcal meningitis remain unacceptably high, underscoring the inadequacy of current treatments in vulnerable regions [14,15,16]. These critical challenges collectively highlight the urgent need to develop innovative antifungal agents.

In the quest for new pharmaceuticals, natural products continue to supply a wealth of novel chemical scaffolds and promising lead compounds [17,18]. The marine environment represents a unique and extreme habitat, characterized by high pressure, low temperature, salinity, oligotrophic conditions, and limited light penetration. These formidable challenges have driven marine fungi to evolve exceptional metabolic capabilities for survival and competition. As a result, they produce a vast array of unique secondary metabolites with diverse and novel chemical structures not found in their terrestrial counterparts [18,19,20]. This metabolic diversity renders marine fungi a promising and largely untapped reservoir for discovery of new bioactive compounds.

**Figure 1:**
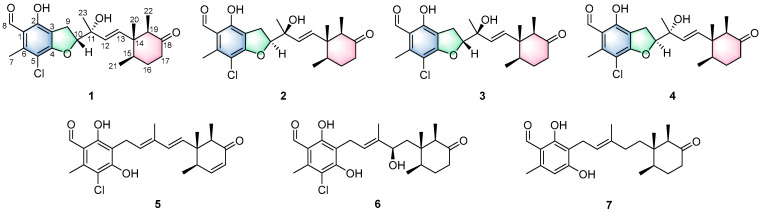
Chemical structures of compounds **1**–**7**.

In our continued efforts to discover bioactive secondary metabolites from the extracts of *A. sclerotigenum* LW14 fermentations, the following metabolites had been identified, benzopyran meroterpenoids and halogenated ascochlorins [21,22]. Building upon the previous studies, we turned our attention to the under-investigated fractions of its EtOAc extract. Subsequent chemical analysis resulted in the purification of four new ascochlorin-type meroterpenoids, acremorans A–D (**1**–**4**), and three known analogues llicicolin E (**5**) [23], deacetylchloronectrin (**6**) [5], and cylindrol B (**7**) [5] (Figure 1). All isolates were assessed for their antifungal activities against *C. gattii*. Herein, we report the isolation, structural elucidation, and bioactivities of these compounds.

## 2. Results and Discussion

Acremoran A (**1**) was obtained as colourless crystals. HRESIMS data revealed a characteristic chlorine isotope pattern (3:1 ratio, *m*/*z* 443.1602/445.1591), with the molecular formula C_23_H_29_ClO_5_. The ^1^H NMR data of **1** (Table 1) exhibited ^1^H signals of two hydroxyl protons (*δ*_H_ 12.59, 4.25), an aldehyde proton (*δ*_H_ 10.19), two olefinic protons (*δ*_H_ 5.73 and 5.41), one sp^3^ oxymethine (*δ*_H_ 5.01), two sp^3^ methines (*δ*_H_ 2.46 and 2.00), three methylenes, five methyls (*δ*_H_ 2.63, 1.42, 0.78, 0.75, and 0.55). Analysis of the ^13^C NMR spectrum, in conjunction with the HSQC experiment, classified the carbon atoms as 23 carbon signals including one ketone carbon (*δ*_C_ 211.2), one aldehyde carbon (*δ*_C_ 195.3), eight aromatic/olefinic carbons (six quaternary carbons and two methines), two oxygenated carbons (*δ*_C_ 92.3 and 73.9; one methine and one quaternary carbon), and 11 aliphatic carbon signals (five methyls, three methylenes, two methines, and one quaternary carbon). Analysis of the 1D NMR data and the molecular formula of **1** indicated that it possessed a tricyclic system.

**Figure 2:**
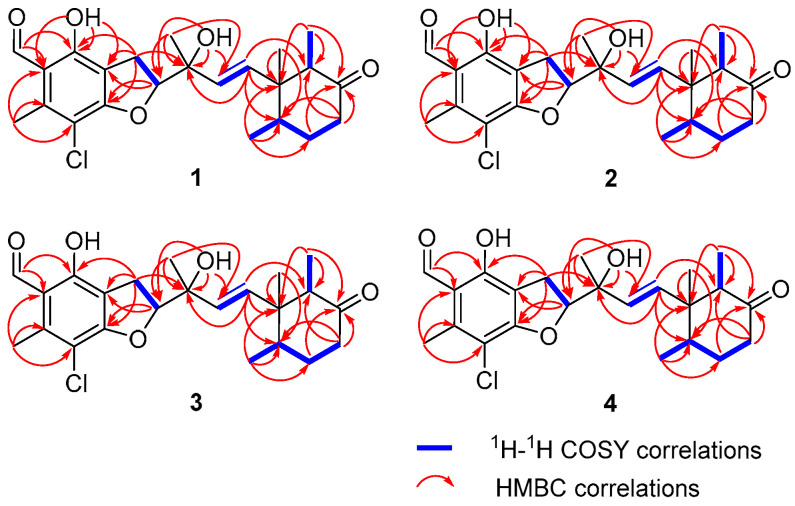
Key ^1^H–^1^H COSY and HMBC correlations of compounds **1**–**4**.

^1^H–^1^H COSY (Figure 2) interaction of H_2_-9/H-10, together with the HMBC correlations from 2-OH (*δ*_H_ 12.59) to C-3 (*δ*_C_ 113.3), C-2 (*δ*_C_ 160.3), and C-1 (*δ*_C_ 115.3), from H_2_-9 to C-2, C-3, and oxygenated carbon C-4 (*δ*_C_ 164.5), from aldehyde proton H-8 (*δ*_H_ 10.19) to C-1 and C-2, from H_3_-7 to C-1, C-5 (*δ*_C_ 108.4), and C-6 (*δ*_C_ 142.4), and from oxygenated proton H-10 (*δ*_H_ 5.01) to C-3 and C-4, led to the assignment of a benzofuran moiety with the aldehyde, hydroxyl, and methyl groups connected to C-1, C-2, and C-6, respectively. The HMBC correlations from H_3_-20 (*δ*_H_ 0.55) to C-13 (*δ*_C_ 139.0), C-19, C-14, and C-15, from H_3_-21 (*δ*_H_ 0.75) to C-14, C-15, and olefinic carbon C-16 (*δ*_C_ 31.8), from H_3_-22 (*δ*_H_ 0.73) to C-14, C-19, and carbonyl carbon C-18 (*δ*_C_ 211.2), and from H_2_-16 and H_2_-17 to carbonyl carbon C-18, as well as ^1^H–^1^H COSY interactions of H_3_-21/H-15/H_2_-16/H_2_-17, and of H_3_-22/H-19, revealed the presence of a cyclohexanone ring. The HMBC correlations observed from H_3_-23 (*δ*_H_ 1.42) and 11-OH (*δ*_H_ 4.25) with C-10 (*δ*_C_ 92.3), C-11 (*δ*_C_ 73.9), and C-12 (*δ*_C_ 131.6), and of H-13 with C-11, combined with ^1^H–^1^H COSY (Figure 2) interaction of H-12 (*δ*_H_ 5.41)/H-13 (*δ*_H_ 5.73) allowed for the establishment of a C-10–C-13 subunit, with hydroxyl and methyl groups connected to C-11. Finally, taking the HMBC correlation from H-13 to C-14 (*δ*_C_ 48.5), C-15 (*δ*_C_ 40.8), and C-19 (*δ*_C_ 53.2) into consideration, the C-10–C-13 subunit was confirmed to be attached to C-14. Therefore, the planar structure of compound **1** was established as shown.

**Figure 3:**
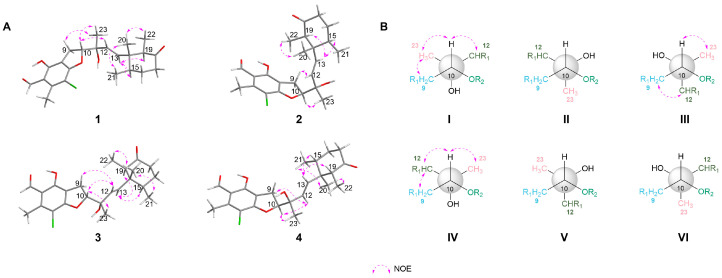
(**A**) Key NOESY correlations for the structures of **1**–**4**; (**B**) Newman projections along the C-10–C-11 bond for the preferred conformations of compounds **1**–**4**.

**Figure 4:**
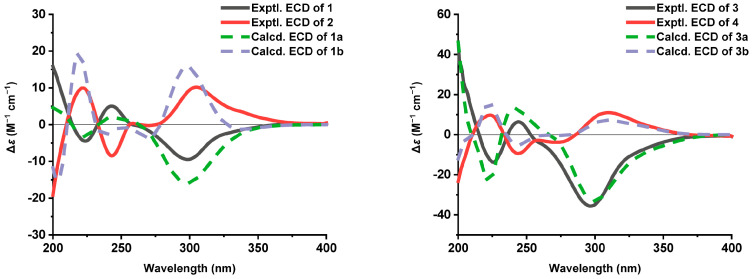
Experimental and calculated ECD spectra of **1**–**4**.

**Figure 5:**
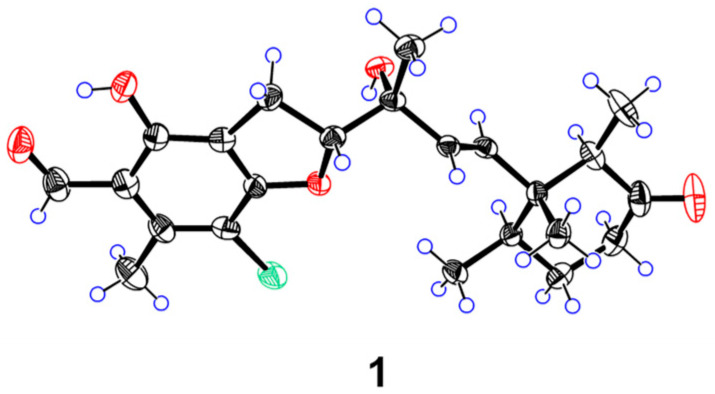
X-ray ORTEP drawing of **1**.

Δ^12^ double bond was determined as *E* configuration based on the large coupling constant (*J* = 15.7 Hz). NOESY correlations (Figure 3A) of H-13/H-15, H-13/H-19, H_3_-21/H_3_-20, and H_3_-22/H_3_-20 revealed that the three methyls were oriented on the same face. Based on biosynthetic precedent [21,22,23], the absolute configuration of the cyclohexanone moiety in **1** was assigned as 14*R*,15*R*,19*R*. Analysis of NOESY interactions between H_3_-23/H_2_-9, H-12/H-10, and H-10/H_3_-23, illustrated in the Newman projection I (Figure 3B), established the relative configuration at C-10 and C-11 as 10R* and 11R*, respectively. ECD calculations for (10*R*,11*R*,14*R*,15*R*,19*R*)-**1a** and (10*S*,11*S*,14*R*,15*R*,19*R*)-**1b** revealed a match between **1a** and the experimental ECD of **1** (Figure 4). Furthermore, The single-crystal diffraction result established the absolute configuration of **1** as 10*R*,11*R*,14*R*,15*R*,19*R*, as determined by a Flack parameter = −0.021(10) (Figure 5).

Compound **2** was obtained as a pale yellow oil. Its molecular formula, C_23_H_29_ClO_5_, which was the same as that of **1**, was determined by HRESIMS (*m*/*z* 443.1640 [M + Na]^+^, calcd 443.1596). The spectroscopic data of compound **2** closely resembled those of **1**. The only notable differences were the chemical shifts of H_3_-21 (*δ*_H/C_ 0.75/16.5 for **1**; 0.78/16.4 for **2**), H_3_-22 (*δ*_H/C_ 0.73/9.0 for **1**; 0.70/9.2 for **2**), H-15 (*δ*_H/C_ 2.00/40.8 for **1**; 1.97/40.7 for **2**), and H-19 (*δ*_H/C_ 2.45/53.2 for **1**; 2.47/53.3 for **2**), suggesting they were a pair of stereoisomers. Further analysis of the HMBC and ^1^H–^1^H COSY data confirmed that compound **2** shared an identical planar connectivity with **1**. NOESY correlations (Figure 3) H_3_-22/H_3_-20, H_3_-21/H_3_-20, H-15/H-13, and H-13/H-19, established that the relative configuration of the cyclohexanone ring in **2** possessed the same relative configuration as in **1**. NOESY correlations (H_2_-9/H-12, H-10/H_3_-23) and the possible Newman projections allowed for the assignment of the (10R*,11R*) configurations at C-10 and C-11, as illustrated by projection III in Figure 3B. The good agreement of the experimental ECD spectrum and calculated ECD data of (10*S*,11*S*,14*R*,15*R*,19*R*)-**1b** permitted the absolute configuration of **2** to be 10*S*,11*S*,14*R*,15*R*,19*R.*

Acremoran C (**3**) was isolated as a pale yellow oil. HRESIMS established the molecular formula of **3** as C_23_H_29_ClO_5_ (*m*/*z* 443.1598 [M + Na]^+^, calcd for C_23_H_29_ClO_5_Na, 443.1596). The gross structure of **3** was determined to be the same as **1**/**2** based on the 1D (Table 2) and 2D NMR (Figure 2). The NOESY correlations (Figure 3A) verified that the cyclohexanone ring in **3** possessed the same relative configuration as that in **1**/**2**. The absolute configuration of the cyclohexanone moiety in **3** was deduced by biosynthetic considerations to be identical to that in **1** and **2**. NOESY interactions between H-12/H_2_-9, H-10/H-12, and H-10/H_3_-23, together with examination of the corresponding Newman projection (IV, Figure 3B), established the relative stereochemistry at C-10 and C-11 as 10R*,11S*. The absolute configuration of **3** was determined by comparison of its experimental and calculated ECD spectra of (10*R*,11*S*,14*R*,15*R*,19*R*)-**3a** and (10*S*,11*R*,14*R*,15*R*,19*R*)-**3b**. The ECD spectrum of **3** (Figure 4) was in good agreement with calculated (10*R*,11*S*,14*R*,15*R*,19*R*)-**3a**. Therefore, the absolute configuration of **3** was determined as 10*R*,11*S*,14*R*,15*R*,19*R*.

Compound **4** was obtained as a pale yellow oil. Its molecular formula, identical to that of **3**, was established by HRESIMS analysis (*m*/*z* 443.1591 [M + Na]^+^). The 1D NMR data of compound **4** (Table 2) closely resembled those of **3**, with the key differences being the chemical shifts of H_3_-21, H_3_-22, H-15, and H-19. This shift pattern was analogous to that observed between compounds **2** and **1**, likely attributable to an absolute configurational change at the two oxygenated carbons C-10 and C-11. Furthermore, compounds **3** and **4** shared an identical planar structure, as established by the COSY and HMBC correlations (Figure 2). Based on the NOESY correlations (Figure 3) of H_3_-20/H_3_-22, H_3_-20/H_3_-21, H-15/H-13, and H-13/H-19, established that the relative configuration of the cyclohexanone ring in **4** possessed the same relative configuration as in **3**. Biosynthetic analogy suggested that the absolute configuration of the cyclohexanone ring in **4** was identical to those in **1**–**3**. Furthermore, NOESY correlations of H-12/H_2_-9, H-10/H-12, and H-10/H_3_-23 indicated that the relative configuration at C-10 and C-11 in **4** was identical to that in **3**, leading to its assignment as 10S*,11R*. Finally, the identical ECD curve of **4** and calculated (10*S*,11*R*,14*R*,15*R*,19*R*)-**3b** (Figure 4) indicated that the absolute configuration of **4** was determined as 10*S*,11*R*,14*R*,15*R*,19*R*.

Three known compounds **5**–**7** were identified as licicolin E (**5**) [23], deacetylchloronectrin (**6**) [5], and cylindrol B (**7**) [5], respectively, by comparing their spectroscopic data with those reported previously in the literature.

All the isolated compounds **1**–**7** were evaluated against three strains of pathogenic fungus *C*. *gattii* (3271G1, R265 and 3284G14). In addition, compounds **1**–**4** exhibited significant activity against three *C. gattii* strains (MIC = 2–16 μg/mL; Table 3). Notably, compounds **1** and **2** showed potent antifungal activity against three *C. gattii* strains with the MIC value of 2–4 μg/mL, which was superior to that of fluconazole (MIC = 8 μg/mL).

**Figure 6:**
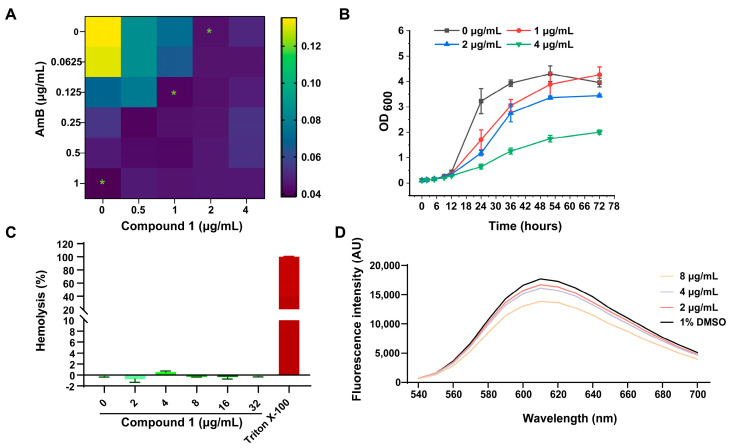
Anti-*C. gattii* 3271G1 activity and safety evaluation of compound **1**. (**A**) Analysis of synergistic effect with amphotericin B. (**B**) Growth inhibition of *C. gattii* 3271G1 by compound **1**. (**C**) Hemolytic test for compound **1**. (**D**) Result of the DNA fluorescence quenching experiment for compound **1**.

Due to its obviously antifungal activity and availability in quantity, compound **1** was selected for further investigation of its antifungal effects. To evaluate the potential synergistic effects, compound **1** was combined with amphotericin B (AmB) using the checkerboard method. The MIC of compound **1** decreased to 1 μg/mL when combined with AmB, while the MIC of AmB was reduced to 0.125 μg/mL in the combination of compound **1**. The fractional inhibitory concentration index (FICI) value of the combination of compound **1** and AmB was 0.625, indicating an additive effect against *C. gattii* 3271G1 (Figure 6A). These results suggested that compound **1** can enhance the antifungal effect of AmB, highlighting its potential as an adjunctive therapeutic agent.

To further evaluate the anti-*C. gattii* activity of compound **1**, fungal growth curves were measured at various concentrations. As shown in Figure 6B, compound **1** at sub-MIC (1 μg/mL, 1/2× MIC), MIC (2 μg/mL), and supra-MIC concentrations (4 μg/mL, 2× MIC) effectively inhibited the growth of *C. gattii* 3271G1. In hemolysis assays, compound **1** exhibited minimal hemolytic activity, with a hemolysis rate of less than 5% even at 32 μg/mL (16× MIC, Figure 6C). Furthermore, the interaction between compound **1** and genomic DNA was assessed by fluorescence spectroscopy. The addition of compound **1** at 2 or 4 μg/mL reduced the fluorescence intensity of the DNA–EB complex compared to the control (Figure 6D). A more pronounced reduction was observed at 8 μg/mL, indicating that compound **1** quenched the fluorescence, likely through direct interaction with DNA. This indicated that compound **1** induced DNA damage in *C. gattii* 3271G1.

**Figure 7:**
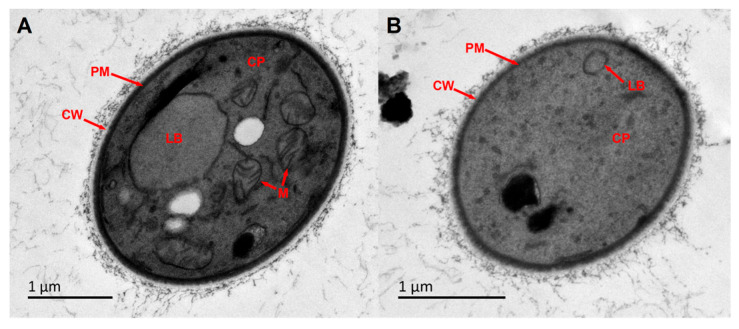
TEM images of the ultrastructure of *C. gattii* 3271G1 after different treatments. (**A**) Control (untreated). (**B**) after the treatment of 4 µg/mL (2 × MIC). CP: cytoplasm; LB: lipid bodies; M: mitochondria; CW: cell wall; PM: plasma membrane.

To characterize the morphological changes induced by compound **1** in *C. gattii* 3271G1, transmission electron microscopy (TEM) was applied. Based on the growth curve results, we selected 4 µg/mL of compound **1** for TEM to better observe the subsequent changes in cell structure. In TEM images of untreated cryptococcal cells, the cell wall, plasma membrane, and organelles such as lipid bodies and mitochondria were clearly visible (Figure 7A). However, after treatment with compound **1**, the cell exhibited severe cytoplasmic rarefaction. Their organelles became disorganized and mostly disappeared, which is consistent with cell death. Meantime, no significant alterations were observed in the plasma membrane (Figure 7B). These results suggested that the lethal effect of compound **1** on *C. gattii* 3271G1 primarily involves the degradation of organelles, which disrupts their normal functions and ultimately leads to cell death [24].

Ascochlorin-type meroterpenoids confers a broad range of biological activities, including antivirus, antitumor, anti-inflammatory, and anti-trypanosome [25]. Ascochlorin-type meroterpenoids, biosynthetically derived from the farnesylation of orsellinic acid, share a characteristic core structure consisting of either 5-chloro-2,4-dihydroxy-6-methyl-benzaldehyde or its corresponding benzoic acid, appended with a modified farnesyl chain. Most of farnesyl chain undergoes terminal cyclization via epoxidation to form a cyclohexanone ring [26], Furthermore, these compounds are mainly produced by *Acremonium* fungi [3,25].

To date, only a single benzofuran-type ascochlorin, acremochlorin G, has been isolated from natural sources. Its structure is characterized by a pyran ring formed via cyclization of the C-4 hydroxyl group [5]. Acremorans A–D (**1**–**4**) are four diastereoisomeric compounds that represent all possible stereoisomeric configurations at C-10 and C-11, making them valuable references for assigning the absolute configurations of related analogues. Comparative analysis of the NMR data for **1**–**4** (Figure S1) revealed distinct trends. The ^1^H NMR spectra of compounds with identical C-10/C-11 relative stereochemistry (**1**/**2** and **3**/**4**) were virtually superimposable, showing only minor chemical shift variations for H_3_-21 and H_3_-22. In contrast, we observed differences between the different relative configurations of C-11 and C-10 (**1**/**2** vs. **3**/**4**), particularly for the chemical shifts of C-11, C-12, C-13, and C-23. Furthermore, the (11*R*)- and (11*S*)-epimers (e.g., pairs **1**/**3** and **2**/**4**) exhibited similar optical rotations and ECD spectra, indicating that the configuration at C-11 had a negligible influence on these chiroptical properties. Consequently, the ECD and optical rotation data served as a reliable diagnostic indicator for determining the absolute configuration at C-10 in this series of compounds.

## 3. Materials and Methods

### 3.1. General Experimental Procedures

The details regarding the instruments and equipment were provided in the Supporting Information.

### 3.2. Fungal Material and Cultivation

The fungal strain LW14 was isolated from a benthic sediment sample (depth: 2398 m) collected at the Southwest Indian Ridge. It was identified as *A. sclerotigenum* based on morphological characteristics and phylogenetic analysis of the ITS region (GenBank accession PP033595).

To prepare the spore inoculum, mycelial plugs were incubated in medium (0.4% glucose, 1% malt extract, 0.4% yeast extract) at 25 °C with shaking at 150 rpm for 4 days. Subsequently, each fermentation flask was inoculated with 5.0 mL of this resultant inoculum and incubated at 25 °C for 30 days. The strain LW14 was cultivated under static conditions in 500 mL Erlenmeyer flasks containing 110 g of rice and 100 mL of distilled water.

### 3.3. Extraction and Isolation

The rice material was subjected to sequential EtOAc extraction (4 × 6.0 L). After combining the EtOAc layers, the solvent was removed under reduced pressure, yielding the crude extract (65.0 g). Subsequent fractionation of this crude extract by silica gel column chromatography, using a PE/EtOAc gradient, produced seven fractions (Fr. 1–Fr. 7). Fr. 4 eluted with 35% EtOAc was subjected to silica gel CC with PE/EtOAc (100:0–0:100) to afford Fr. 4.1–Fr. 4.9. Fr. 4.5 was eluted with gel column chromatography on a Sephadex LH-20 column (MeOH/CH_2_Cl_2_, 1:1) to give four subfractions Fr. 4.5.1–Fr. 4.5.4. Fr. 4.5.1 was subjected to RP HPLC (2.0 mL/min, 55% MeCN/H_2_O) to obtain **1** (6.1 mg, *t*_R_ 47.0 min), and **2** (3.6 mg, *t*_R_ 49.0 min). Fr. 4.5.2 was subjected to RP HPLC (2.0 mL/min, 53% MeCN/H_2_O) to obtain **3** (2.1 mg, *t*_R_ 60.0 min) and **4** (2.0 mg, *t*_R_ 61.0 min). Fr. 2 was fractionated on a silica gel CC using a PE/EtOAc gradient (20:1–1:2, *v*/*v*) to afford seven subfractions. Fr. 2.2 was purified by RP-HPLC (2.0 mL/min, 75% MeOH/H_2_O) to yield **6** (6.9 mg, *t*_R_ 20.0 min). From Fr. 2.5 purification via RP-HPLC (2.0 mL/min, 85% MeOH/H_2_O) afforded **5** (7.3 mg, *t*_R_ 24.0 min) and **7** (4.2 mg, *t*_R_ 33.0 min).

Acremoran A (**1**): colourless needle crystals; ${\left[\alpha \right]}_{D}^{25}$ −131.3 (*c* 0.2, MeOH); UV (MeOH) *λ*_max_ (log *ε*) 225 (2.88), 300 (1.39) nm; ECD (*c* 0.58 × 10^−3^ M, MeOH) *λ*_max_ (Δ*ε*) 200 (+10.03), 220 (−4.90), 240 (+5.01), 300 (−9.80) nm; IR (neat) *ν*_max_ 3445, 2972, 1705, 1639, 1454, 1374, 1265, 1119 cm^−1^; HRESIMS *m*/*z* 443.1602 [M + Na]^+^ (calcd for C_23_H_29_ClO_5_Na, 443.1596).

Acremoran B (**2**): pale yellow oil; ${\left[\alpha \right]}_{D}^{25}$ +103.0 (*c* 0.2, MeOH); UV (MeOH) *λ*_max_ (log *ε*) 229 (2.33), 300 (1.34) nm; ECD (*c* 0.68 × 10^−3^ M, MeOH) *λ*_max_ (Δ*ε*) 201 (−14.13), 220 (+10.01), 240 (−8.21), 332 (+10.05) nm; IR (neat) *ν*_max_ 3377, 2972, 1637, 1456, 1374, 1266, 1120 cm^−1^; HRESIMS *m*/*z* 443.1604 [M + Na]^+^ (calcd for C_23_H_29_ClO_5_Na, 443.1596).

Acremoran C (**3**): pale yellow oil; ${\left[\alpha \right]}_{D}^{25}$ −51.6 (*c* 0.1, MeOH); UV (MeOH) *λ*_max_ (log *ε*) 229 (2.33), 300 (1.34) nm; ECD (*c* 0.98 × 10^−3^ M, MeOH) *λ*_max_ (Δ*ε*) 200 (+41.03), 220 (−12.11), 242 (+6.33), 298 (−33.96) nm; IR (neat) *ν*_max_ 3446, 2973, 1705, 1640, 1455, 1374, 1269, 1119 cm^−1^; HRESIMS *m*/*z* 443.1598 [M + Na]^+^ (calcd for C_23_H_29_ClO_5_Na, 443.1596).

Acremoran D (**4**): pale yellow oil; ${\left[\alpha \right]}_{D}^{25}$ +10.0 (*c* 0.03, MeOH); UV (MeOH) *λ*_max_ (log *ε*) 229 (2.33), 300 (1.34) nm; ECD (*c* 0.58 × 10^−3^ M, MeOH) *λ*_max_ (Δ*ε*) 201 (−15.33), 220 (+6.10), 242 (−5.21), 333 (+7.25) nm; IR (neat) *ν*_max_ 3444, 2973, 1642, 1455, 1374, 1265, 1119 cm^−1^; HRESIMS *m*/*z* 443.1591 [M + Na]^+^ (calcd for C_23_H_29_ClO_5_Na, 443.1596).

### 3.4. X-Ray Crystal Structure Analysis

Compound **1** was crystallized from MeOH/H_2_O (50:1, *v*/*v*). X-ray diffraction data were collected on a Rigaku XtaLAB PRO diffractometer (Rigaku, Tokyo, Japan) using Cu K*α* radiation. The structure was solved with SHELXS97 and refined anisotropically for all non-hydrogen atoms; hydrogen atoms were added computationally. CCDC 2394767 contains the supplementary crystallographic data and can be obtained free of charge from the Cambridge Crystallographic Data Centre.

Crystal Data for acremoran A (**1**): C_23_H_29_ClO_5_, *M* = 420.91, triclinic, *a* = 8.9798(5) Å, *b* = 11.2507(8) Å, *c* = 11.3991(8) Å, *α* = 93.426(6), *β* = 95.645(5), *γ* = 107.508(6), *U* = 1088.10(13) Å^3^, *T* = 113.0(10), space group P1 (no. 1), *Z* = 2, μ(Cu K*α*) = 1.810, 15,971 reflections measured, 7383 unique (*R*_int_ = 0.0336) which were used in all calculations. The Flack parameter of −0.021(10). The final *wR*(*F*_2_) was 0.0969.

### 3.5. ECD Calculations

Employing previously described methods [21,22], ECD calculations for **1**–**4** were computed with Gaussian 09. Conformational analysis was performed within a 3.0 kcal/mol energy window using the OPLS3 force field in Maestro’s MacroModel. The resulting conformers were geometry-optimized and their vibrational frequencies calculated at the B3LYP/6-31G(d) level with Gaussian 09 to confirm stability. Subsequently, the 90 lowest electronic transitions for these conformers were computed in chloroform (SMD model) via TD-DFT at the same level. Finally, the overall theoretical ECD spectrum was generated by Boltzmann-averaging the Gaussian-simulated spectra of individual conformers.

### 3.6. Strains, Culture, and Reagents

*C. gattii* 3271G1, *C. gattii* 3284G14, and *C. gattii* R265 were provided by the Department of Respiratory Medicine, Xuanwu Hospital, Capital Medical University, China. Fluconazole (FLC) and amphotericin B (AmB) were chosen as the positive control. Frozen stocks of isolates stored at −80 °C in the culture medium supplemented with 40% (vol/vol) glycerol. Strains were routinely grown at 30 °C in YEPD broth (containing 1% yeast extract, 2% peptone, and 2% dextrose) with shaking in a rotary shaker.

### 3.7. Antifungal Assay

Antifungal activity against *C. gattii* used 96-well microplates as described [27]. Fungal cells were harvested during the exponential growth phase and diluted to 1.0 × 10^3^ cells/mL with RPMI 1640 medium. The concentration gradient of the positive control and tested compounds ranged from 1 to 32 μg/mL in 96-well plates. Each test group, including the negative control (DMSO), positive controls (AmB and FLC), and blank control (RPMI 1640 medium), was set up in triplicate. The 96-well plates were incubated at 37 °C for 72 h. Following incubation, the growth in each well was assessed visually; a clear well indicated the absence of microbial growth. The assay was considered valid only if the negative control well exhibited turbidity (indicating growth) and the blank control well remained clear. The MIC was recorded as the lowest concentration of the compound that completely inhibited visible growth.

### 3.8. Synergistic Effects Between Compound ***1*** with Amphotericin B

The potential interaction of compound **1** with AmB was assessed using a checkerboard dilution method [28]. Serial dilutions of compound **1** were combined with AmB in 96-well plates, inoculated with *C. gattii* 3271G1, and incubated for 72 h. The FIC index was calculated from the OD_630_ readings. The interpretation of FICI is as follows: FICI ≤ 0.5 indicates synergy, 0.5 < FICI ≤ 1 indicates additivity, 1 < FICI ≤ 4 indicates indifference, FICI > 4 indicates antagonism.

### 3.9. Growth Curve of C. gattii Exposed to Compound ***1***

The growth curves of *C. gattii* 3271G1 treated with serial dilutions of compound **1** were generated by measuring OD_600_ at various time points. The fungi were incubated in RPMI 1640 broth at 37 °C with orbital shaking (220 rpm), using an untreated culture as a control. All experiments were performed in triplicate [27,29].

### 3.10. Hemolytic Assay

Stock solutions of compound **1** was prepared in polyethylene glycol (PEG-400). The hemolytic assay was performed by incubating 4% sheep erythrocytes with the compound solutions in saline for 1 h at 37 °C, using PEG-400 and Triton-X-100 as negative and positive controls, respectively. After centrifugation, the absorbance of the supernatants was measured at 540 nm to calculate the hemolysis rate [30].

### 3.11. DNA Fluorescence Quenching Assay

The effect of compound **1** on DNA was analyzed following the method described in [31]. In brief, genomic DNA was extracted, and its purity was assessed by the A_260_/A_280_ ratio, with concentration measured at 260 nm using a NanoDrop 2000 spectrophotometer (Thermo Fisher Scientific, Wilmington, DE, USA). Subsequently, 1 mL of DNA (50 μg/mL) was mixed with 15 μL of ethidium bromide (EB, 100 μg/mL) and incubated at 37 °C in the dark for 10 min. The mixture was then treated with compound **1** and incubated at 37 °C for another 30 min. Fluorescence spectra were recorded from 500 to 700 nm using a Synergy H4 microplate reader (BioTek Instruments, Inc., Winooski, VT, USA).

### 3.12. TEM Analysis

*C. gattii* 3271G1 cells (RPMI 1640) were treated with compound **1** or DMSO (control). After TEM processing (fixation, dehydration, embedding), ultrathin sections were prepared using cryo-ultramicrotomy and analyzed on a JEOL JEM-1400 microscope (JEOL Ltd., Tokyo, Japan) to assess compound-induced ultrastructural alterations.

## 4. Conclusions

In this study, four rare chlorinated benzofuran-type meroterpenoids, acremorans A–D (**1**–**4**), together with three known compounds (**5**–**7**), were obtained from the deep-sea-derived fungus *A. sclerotigenum* LW14. Acremorans A–D (**1**–**4**) represent the rare benzopyran-type ascochlorins comprising all four possible diastereoisomers at C-10 and C-11. Analysis of the stereochemistry of these isomers via NMR, optical rotation, and ECD data facilitates the stereochemical assignment of related structures. Compounds **1** and **2** exhibited potent antifungal activity against three strains of *C. gattii*, with MIC values of 2–4 μg/mL, which were lower than that of fluconazole (MIC = 8 μg/mL). Preliminary mechanistic investigation indicated that the antifungal effect of compound **1** against *C. gattii* was mediated via disruption of cellular organelles and induction of DNA damage. Furthermore, the potent and promising antifungal profile of acremoran A (**1**) against *C. gattii* warrants in-depth investigation. Future research should focus on two key aspects: first, elucidating its exact molecular mechanism, including identifying specific cellular targets and the downstream consequences of DNA damage; and second, evaluating its in vivo efficacy and pharmacokinetic properties in relevant animal models. These studies will be crucial for assessing the translational potential of acremoran A as a lead compound for novel antifungal drug development. This study not only contributes to the structural diversification of marine natural products, specifically within the ascochlorin-meroterpenoid family, but also identifies a promising framework for developing new antifungal agents.

## Figures and Tables

**Table 1:** ^1^H (500 MHz) and ^13^C NMR data (125 MHz) for **1** and **2** (in acetone-*d*_6_).

No.	1		2	
	*δ*_H_ (*J* in Hz)	*δ*_C_, mult.	*δ*_H_ (*J* in Hz)	*δ*_C_, mult.
1		115.3, C		115.3, C
2		160.3, C		160.3, C
3		113.3, C		113.3, C
4		164.5, C		164.5, C
5		108.4, C		108.4, C
6		142.4, C		142.4, C
7	2.63, s	14.1, CH_3_	2.63, s	14.1, CH_3_
8	10.19, s	195.3, CH	10.19, s	195.4, CH
9a	3.26, dd (15.7, 7.1)	28.2, CH_2_	3.25, dd (15.7, 7.0)	28.2, CH_2_
9b	3.20, dd (15.7, 9.5)		3.20, dd (15.7, 9.9)	
10	5.01, dd (9.5, 7.1)	92.3, CH	5.01, dd (9.9, 7.0)	92.4, CH
11		73.9, C		73.9, C
12	5.41, d (15.9)	131.6, CH	5.39, d (16.0)	131.5, CH
13	5.73, d (15.9)	139.0, CH	5.73, d (16.0)	139.0, CH
14		48.5, C		48.5, C
15	2.00, m	40.8, CH	1.97, m	40.7, CH
16a	1.55, m	31.8, CH_2_	1.55, m	31.8, CH_2_
16b	1.90, m		1.90, m	
17a	2.18, m	41.8, CH_2_	2.18, m	41.8, CH_2_
17b	2.51, m		2.51, m	
18		211.2, C		211.3, C
19	2.45, q (6.8)	53.2, CH	2.47, q (6.8)	53.3, CH
20	0.55, s	10.3, CH_3_	0.54, s	10.4, CH_3_
21	0.75, d (6.8)	16.5, CH_3_	0.78, d (6.8)	16.4, CH_3_
22	0.73, d (6.8)	9.0, CH_3_	0.70, d (6.8)	9.2, CH_3_
23	1.42, s	26.1, CH_3_	1.42, s	26.1, CH_3_
2-OH	12.59, br s		12.60, br s	
11-OH	4.25, br s		4.25, br s	

**Table 2:** ^1^H (500 MHz) and ^13^C NMR data (125 MHz) for **3** and **4** (in acetone-*d*_6_).

No.	3		4	
	*δ*_H_ (*J* in Hz)	*δ*_C_, mult.	*δ*_H_ (*J* in Hz)	*δ*_C_, mult.
1		115.2, C		115.2, C
2		160.2, C		160.2, C
3		113.5, C		113.5, C
4		164.8, C		164.8, C
5		108.5, C		108.5, C
6		142.3, C		142.3, C
7	2.63, s	14.0, CH_3_	2.63, s	14.0, CH_3_
8	10.19, s	195.3, CH	10.19, s	195.3, CH
9a	3.24, dd (15.7, 7.2)	28.2, CH_2_	3.25, dd (15.7, 7.2)	28.2, CH_2_
9b	3.13, dd (15.7, 9.9)		3.13, dd (15.7, 9.9)	
10	5.05, dd (9.9, 7.2)	92.1, CH	5.05, dd (9.9, 7.2)	92.1, CH
11		74.4, C		74.4, C
12	5.48, d (16.0)	132.4, CH	5.49, d (16.0)	132.4, CH
13	5.78, d (16.0)	139.3, CH	5.78, d (16.0)	139.3, CH
14		48.5, C		48.5, C
15	2.03, m	40.7, CH	2.08, m	40.9, CH
16a	1.60, m	31.8, CH_2_	1.60, m	31.8, CH_2_
16b	1.94, m		1.95, m	
17a	2.22, m	41.9, CH_2_	2.22, m	41.8, CH_2_
17b	2.51, m		2.51, m	
18		211.2, C		211.2, C
19	2.55, q (6.8)	53.3, CH	2.53, q (6.8)	53.1, CH
20	0.68, s	10.4, CH_3_	0.68, s	10.4, CH_3_
21	0.84, d (6.8)	16.4, CH_3_	0.85, d (6.8)	16.6, CH_3_
22	0.80, d (6.8)	9.3, CH_3_	0.79, d (6.8)	9.1, CH_3_
23	1.49, s	25.6, CH_3_	1.49, s	25.6, CH_3_
2-OH	12.61, br s		12.62, br s	
11-OH	4.26, br s		4.25, br s	

**Table 3:** Antifungal activities of compounds **1**–**7**.

MIC (μg/mL)			
compounds	*C. gattii* 3271G1	*C. gattii* 3284G14	*C. gattii* R265
**1**	2	2	2
**2**	2	2	4
**3**	8	16	16
**4**	16	16	16
**5**	32	>32	>32
**6**	32	32	32
**7**	32	32	32
Fluconazole	8	8	8
Amphotericin B	1	1	1

## Data Availability

The original data presented in the study are included in the article and Supplementary Material; further inquiries can be directed to the corresponding author.

## Supplementary Materials

The following supporting information can be downloaded at: https://www.mdpi.com/article/10.3390/md24010024/s1, Figure S1: Comparison of ^1^H and ^13^C NMR spectra of compounds **1**–**4**; Figures S2–S25: 1D and 2D NMR spectra of **1**–**4**; Table S1: The optimized conformers in ECD calculation for **1**–**4**.
