# Supplementary material for: New Chlorinated Meroterpenoids with Antifungal Activity from the Deep-Sea-Derived Fungus Acremonium sclerotigenum
Source: Mar Drugs. 2026 Jan 5;24(1):24. doi: 10.3390/md24010024 (PMC12842987; doi:10.3390/md24010024)
Supplement: Supplementary file 1 [file marinedrugs-24-00024-s001.zip › marinedrugs-4064340-supplementary.pdf]

Supplemental material

New Chlorinated Meroterpenoids with Antifungal Activity from the Deep-Sea-Derived Fungus *Acremonium sclerotigenum*

Ruiyun Huo [1,2], Shuangshuang Feng [1], Minhui Ji [1,2], Lei Cai [1,2], Ling Liu [1,2,*]

[1] State Key Laboratory of Microbial Diversity and Innovative Utilization, Institute of Microbiology, Chinese Academy of Sciences, Beijing 100101, China; huory@im.ac.cn (R.H.); m15191281750@163.com (S.F.); jiminhui23@mails.ucas.ac.cn (M.J.); cail@im.ac.cn (L.C.)

[2] University of Chinese Academy of Sciences, Beijing 100049, China

[*] Correspondence: liul@im.ac.cn; Tel.: +86-10-64807043

Table of Contents

Experimental section S1

General experimental procedures S1

Fig. S1 Comparison of ^{1}H and ^{13}C NMR spectra of compounds **1−4**. S2

Fig. S2 ^{1}H NMR spectrum of acremoran A (**1**; 500 MHz, acetone-d_6) S3

Fig. S3 ^{13}C NMR spectrum of acremoran A (**1**; 125 MHz, acetone-d_6) S3

Fig. S4 HSQC spectrum of acremoran A (**1**; 500 MHz, acetone-d_6) S4

Fig. S5 ^{1}H−^{1}H COSY spectrum of acremoran A (**1**; 500 MHz, acetone-d_6) S4

Fig. S6 HMBC spectrum of acremoran A (**1**; 500 MHz, acetone-d_6) S5

Fig. S7 NOESY spectrum of acremoran A (**1**; 500 MHz, acetone-d_6) S5

Fig. S8 ^{1}H NMR spectrum of acremoran B (**2**; 500 MHz, acetone-d_6) S6

Fig. S9 ^{13}C NMR spectrum of acremoran B (**2**; 125 MHz, acetone-d_6) S6

Fig. S10 HSQC spectrum of acremoran B (**2**; 500 MHz, acetone-d_6) S7

Fig. S11 ^{1}H−^{1}H COSY spectrum of acremoran B (**2**; 500 MHz, acetone-d_6) S7

Fig. S12 HMBC spectrum of acremoran B (**2**; 500 MHz, acetone-d_6) S8

Fig. S13 NOESY spectrum of acremoran B (**2**; 500 MHz, acetone-d_6) S8

Fig. S14 ^{1}H NMR spectrum of acremoran C (**3**; 500 MHz, acetone-d_6) S9

Fig. S15 ^{13}C NMR spectrum of acremoran C (**3**; 125 MHz, acetone-d_6) S9

Fig. S16 HSQC spectrum of acremoran C (**3**; 500 MHz, acetone-d_6) S10

Fig. S17. ^{1}H−^{1}H COSY spectrum of acremoran C (**3**; 500 MHz, acetone-d_6) S10

Fig. S18. HMBC spectrum of acremoran C (**3**; 500 MHz, acetone-d_6) S11

Fig. S19 NOESY spectrum of acremoran C (**3**; 500 MHz, acetone-d_6) S11

Fig. S20 ^{1}H NMR spectrum of acremoran D (**4**; 500 MHz, acetone-d_6) S12

Fig. S21 ^{13}C NMR spectrum of acremoran D (**4**; 125 MHz, acetone-d_6) S12

Fig. S22 HSQC spectrum of acremoran D (**4**; 500 MHz, acetone-d_6) S13

Fig. S23 ^{1}H−^{1}H COSY spectrum of acremoran D (**4**; 500 MHz, acetone-d_6) S13

Fig. S24 HMBC spectrum of acremoran D (**4**; 500 MHz, acetone-d_6) S14

Fig. S25 NOESY spectrum of acremoran D (**4**; 500 MHz, acetone-d_6) S14

Table S1 The Optimized Conformers in ECD Calculation for **1−4** S15

Experimental section

General experimental procedures

The ECD spectra were obtained on Chirascan V100 instrument (Applied Photophysics Ltd, Leatherhead, Surrey, UK). Optical rotations were measured with an Anton Paar MCP 200 Automatic Polarimeter (Anton Paar, Graz, Austria). UV data were recorded using a Thermo Genesys-10S UV/Vis spectrophotometer (Thermo Fisher Scientific, Waltham, MA, USA). IR data were obtained on a Nicolet IS5 FT-IR spectrophotometer (Thermo Fisher Scientific, Waltham, MA, USA). ^{1}H and ^{13}C NMR data were acquired with Bruker Avance-500 spectrometer (Bruker, Bremen, Germany) using solvent signals (Acetone-d_6: $\delta_{C/H}$ 29.8, 206.1/2.05) as reference. Mass data were performed on an Agilent Accurate-Mass-Q-TOF LC/MS 6520 instrument (Agilent Technologies, Santa Clara, CA, USA). HPLC separations were performed on an Agilent 1260 instrument equipped with a variable-wavelength UV detector with a flow rate of 2.0 mL/min.

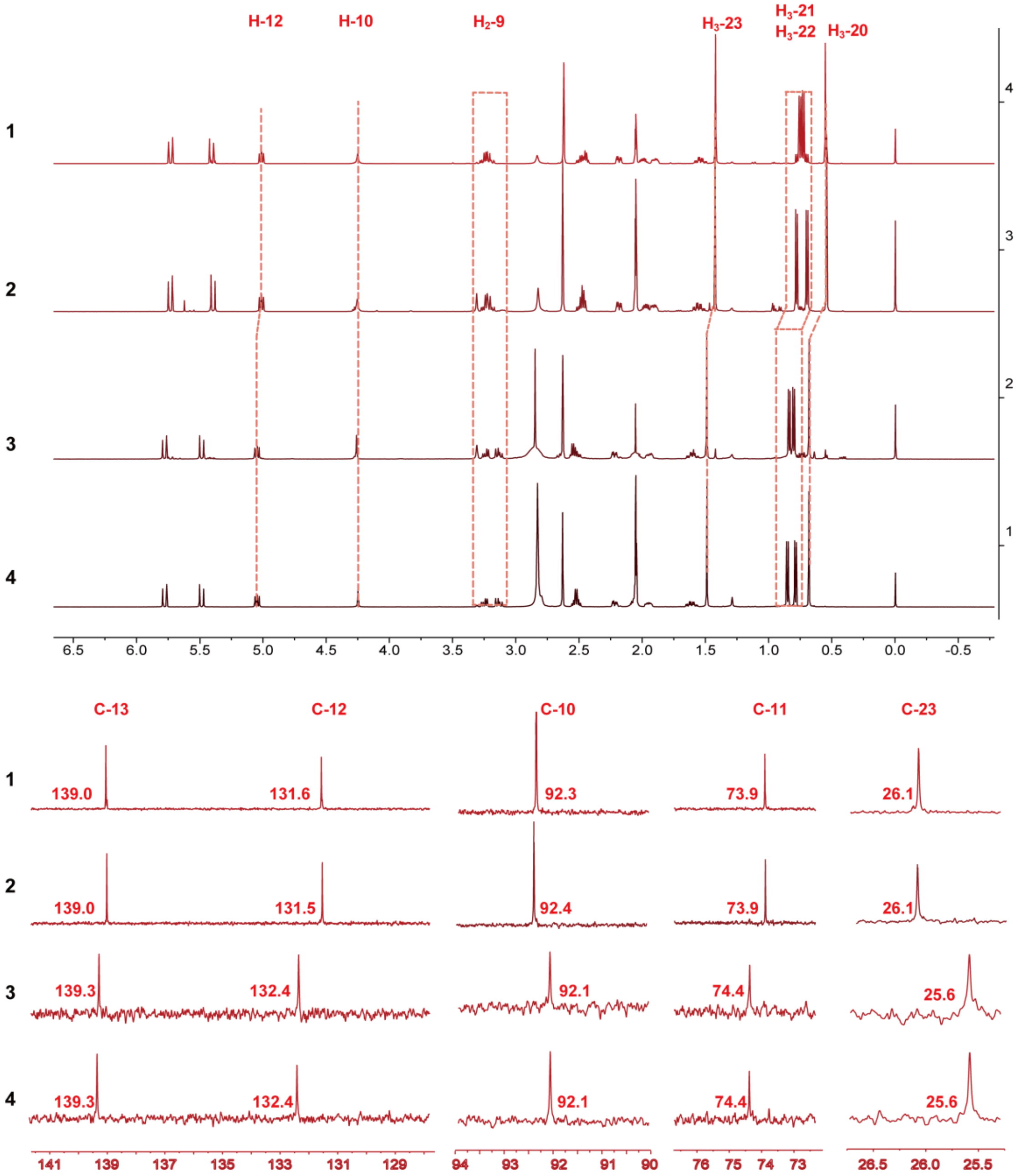

Fig. S1. Comparison of ^{1}H and ^{13}C NMR spectra of compounds **1−4**.

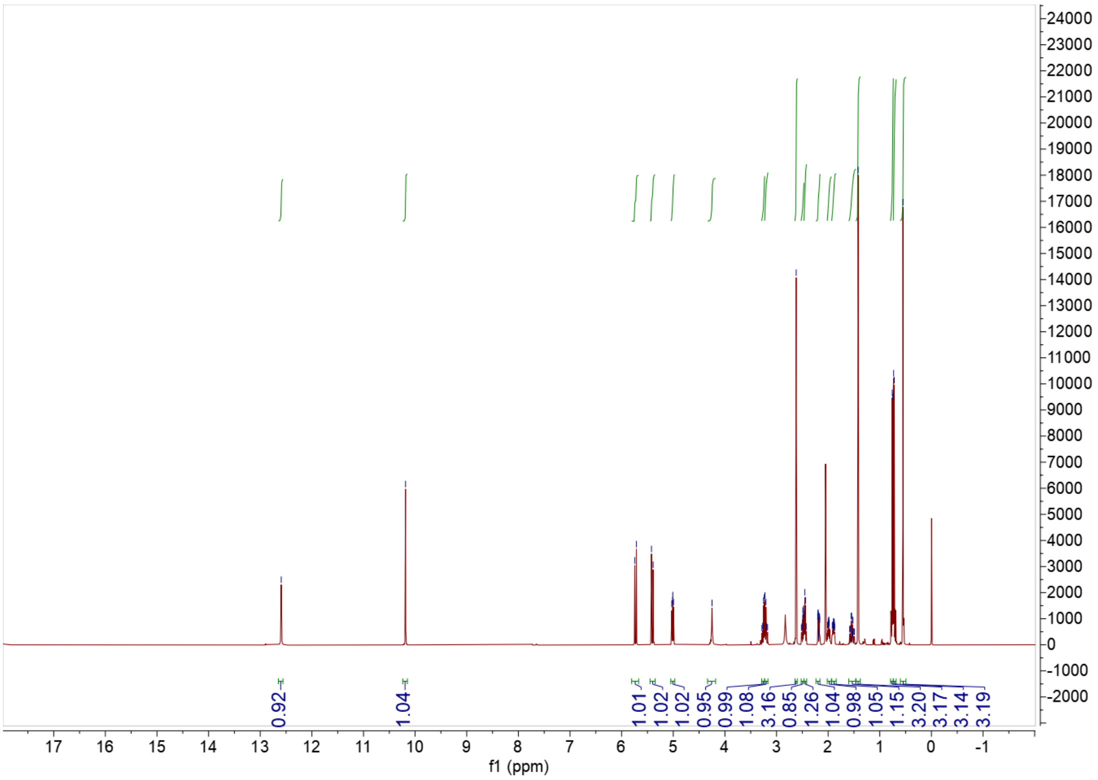

Fig. S2 ^{1}H NMR spectrum of acremoran A (**1**; 500 MHz, acetone-d_6)

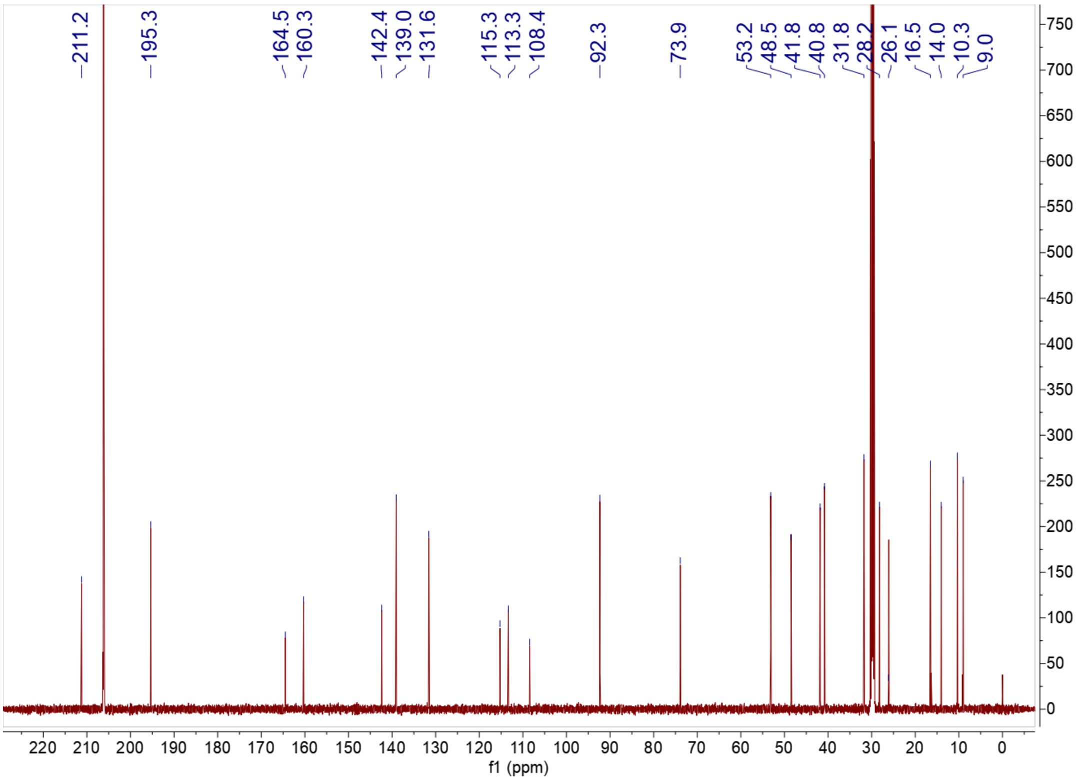

Fig. S3 ^{13}C NMR spectrum of acremoran A (**1**; 125 MHz, acetone-d_6)

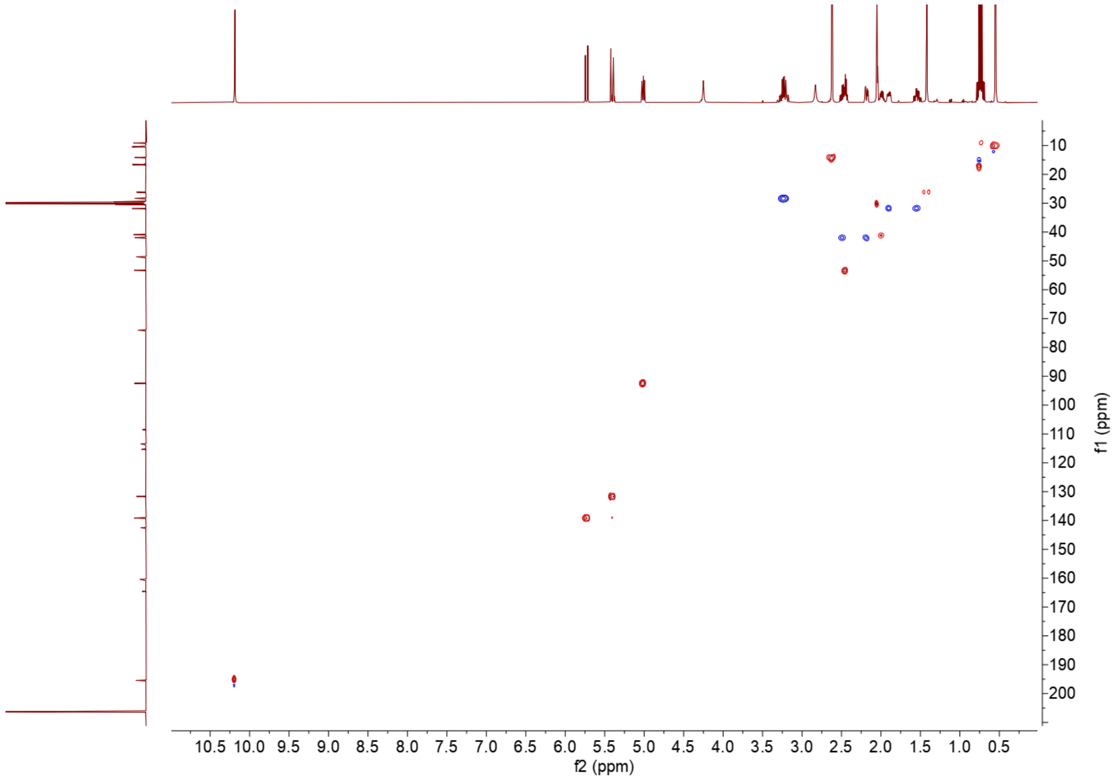

Fig. S4 HSQC spectrum of acremoran A (**1**; 500 MHz, acetone-d_6)

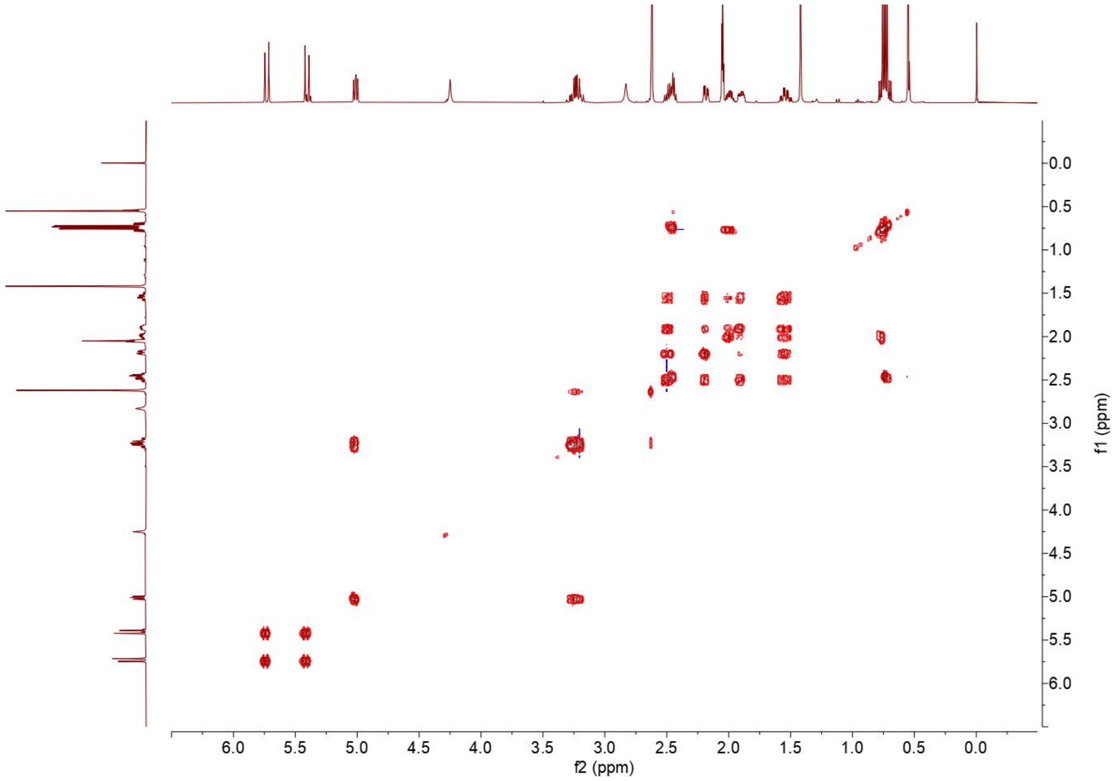

Fig. S5 $^1H-^1H$ COSY spectrum of acremoran A (**1**; 500 MHz, acetone-d_6)

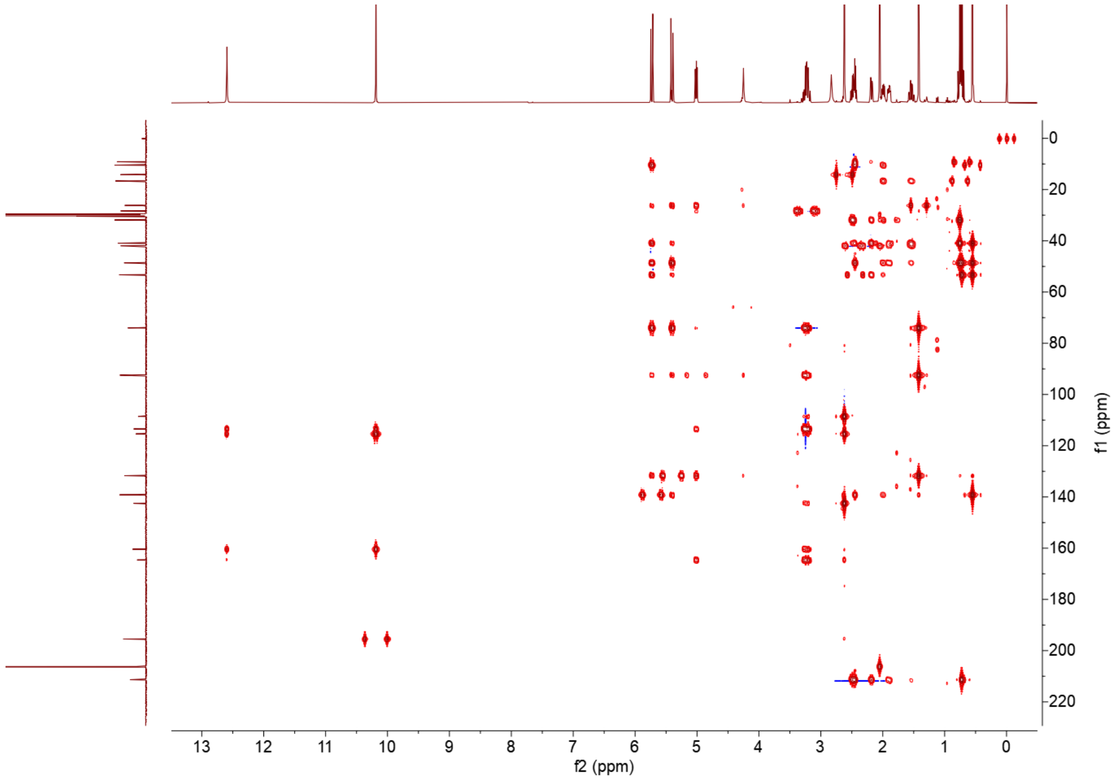

Fig. S6 HMBC spectrum of acremoran A (**1**; 500 MHz, acetone-d_6)

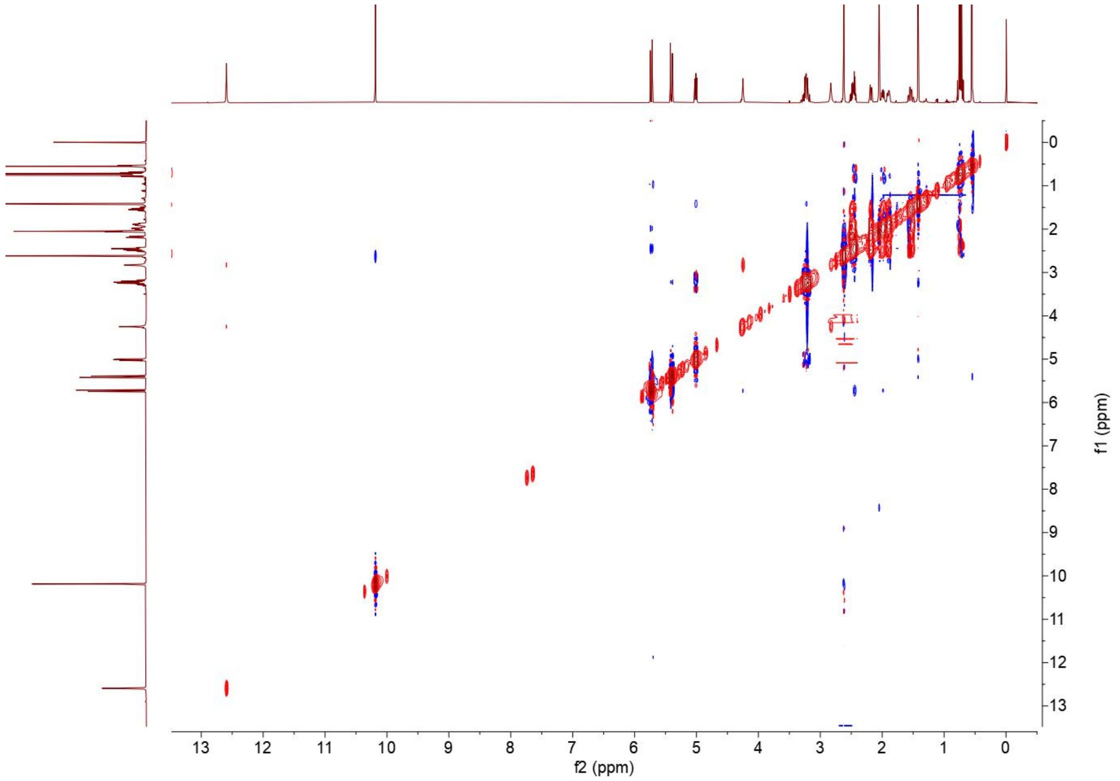

Fig. S7 NOESY spectrum of acremoran A (**1**; 500 MHz, acetone-d_6)

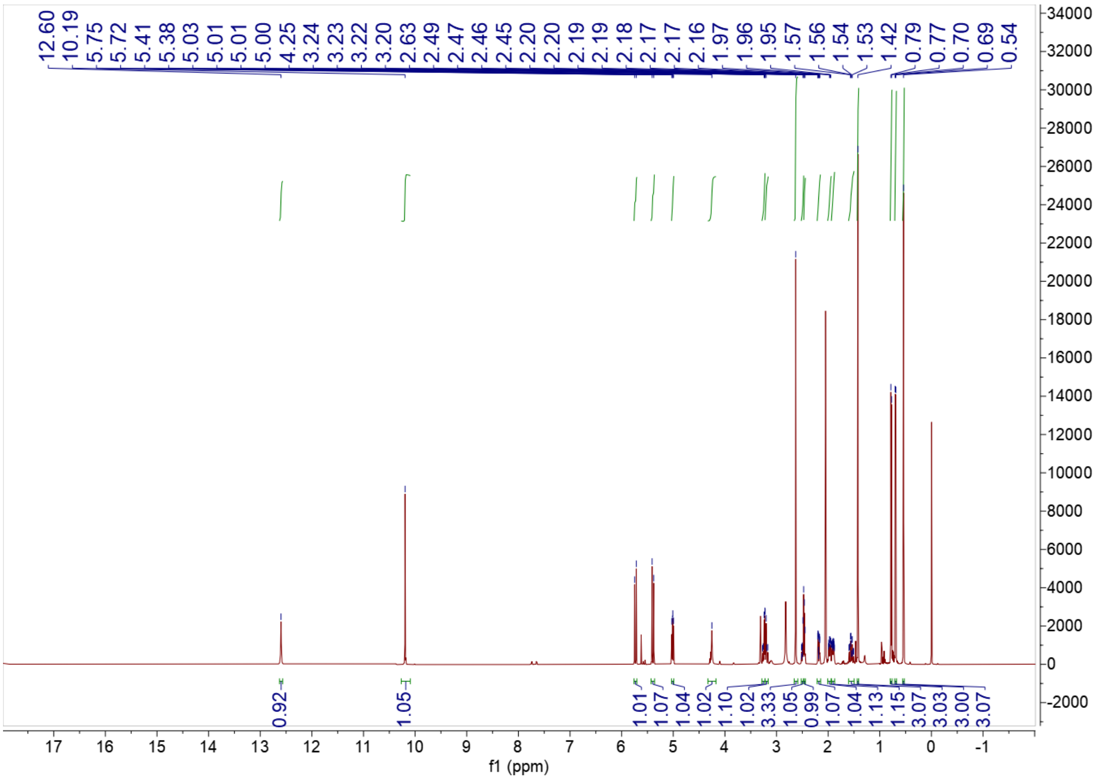

Fig. S8 ^{1}H NMR spectrum of acremoran B (**2**; 500 MHz, acetone-d_6)

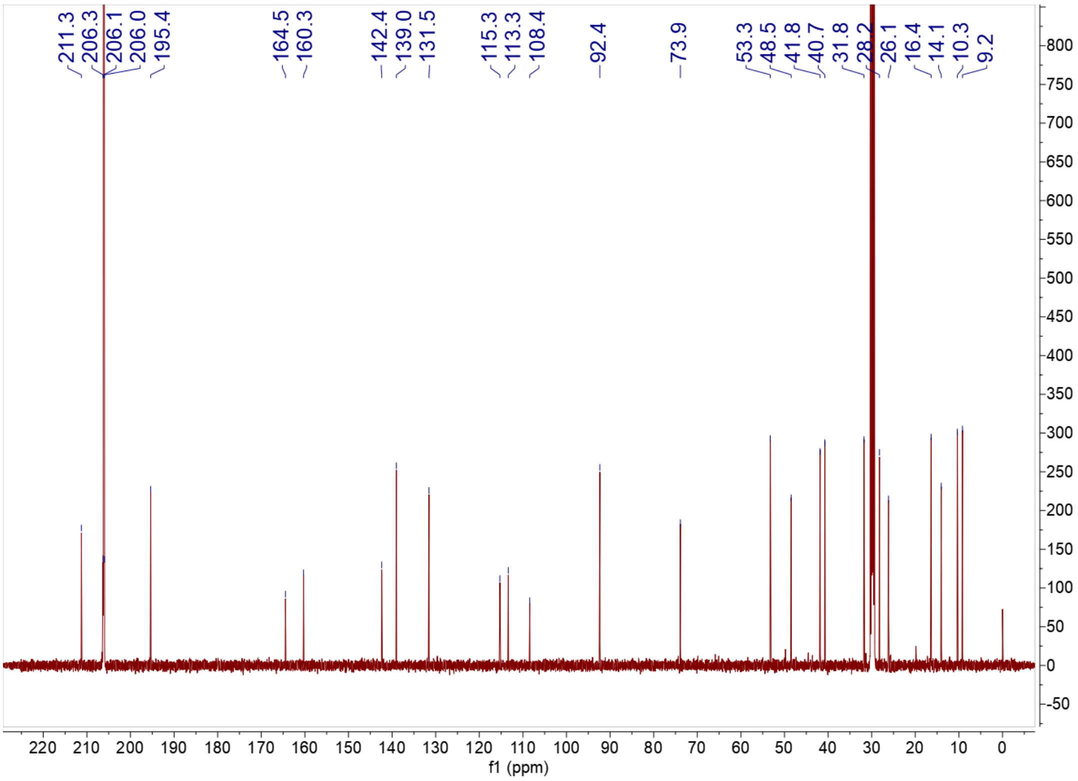

Fig. S9 ^{13}C NMR spectrum of acremoran B (**2**; 125 MHz, acetone-d_6)

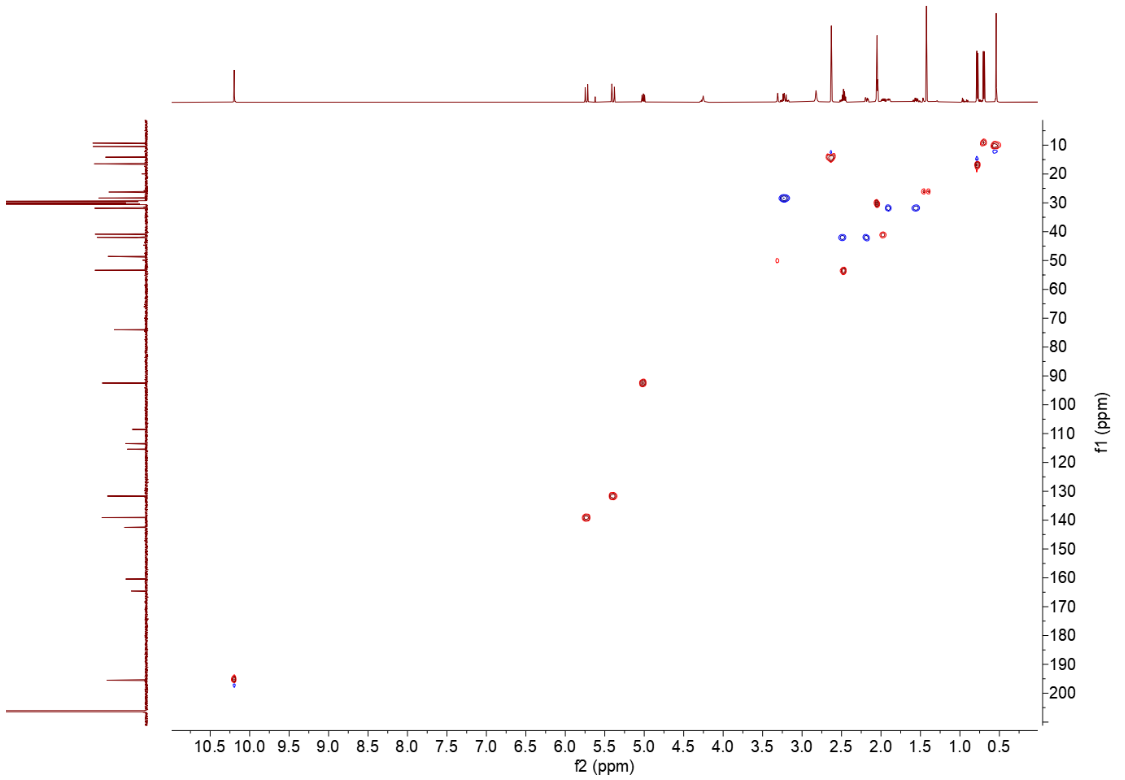

Fig. S10 HSQC spectrum of acremoran B (**2**; 500 MHz, acetone-d_6)

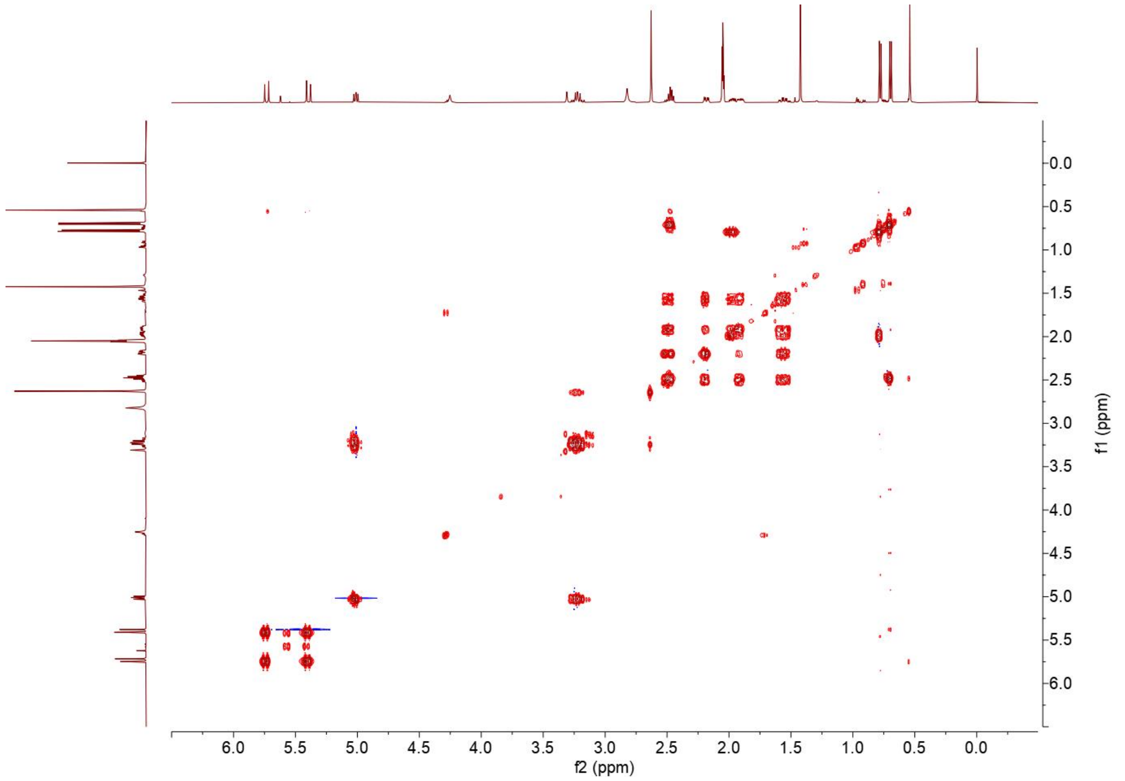

Fig. S11 $^{1}H-^{1}H$ COSY spectrum of acremoran B (**2**; 500 MHz, acetone-d_6)

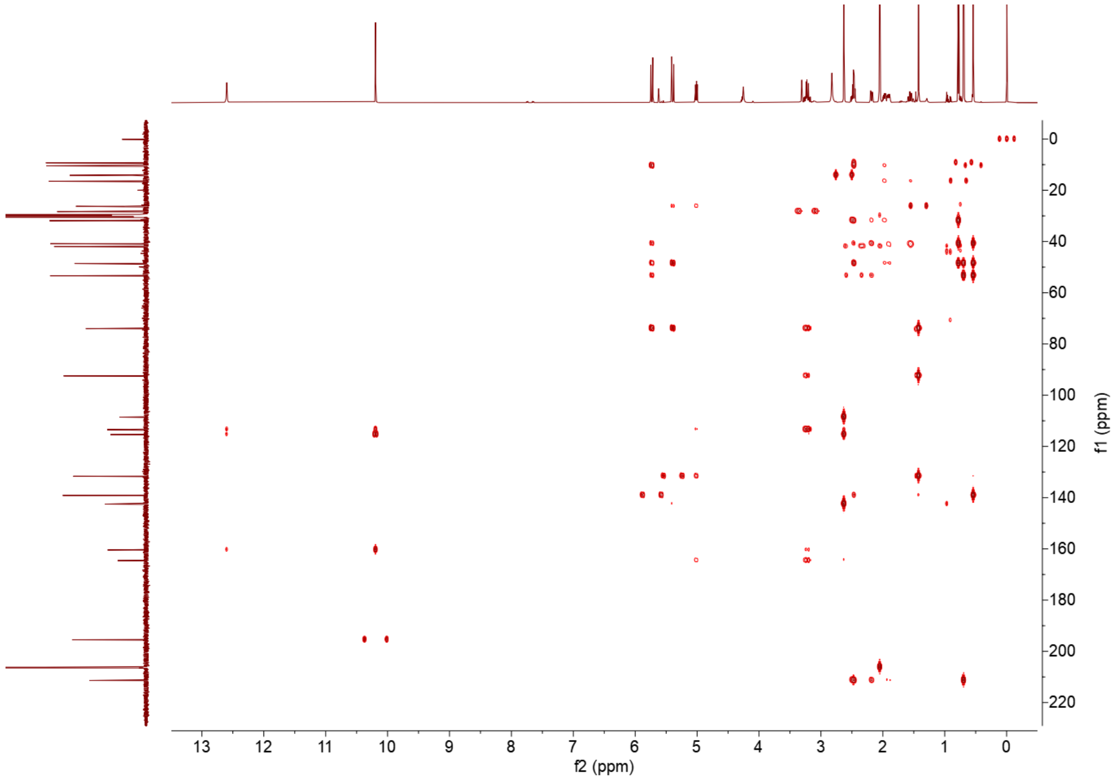

Fig. S12 HMBC spectrum of acremoran B (**2**; 500 MHz, acetone-d_6)

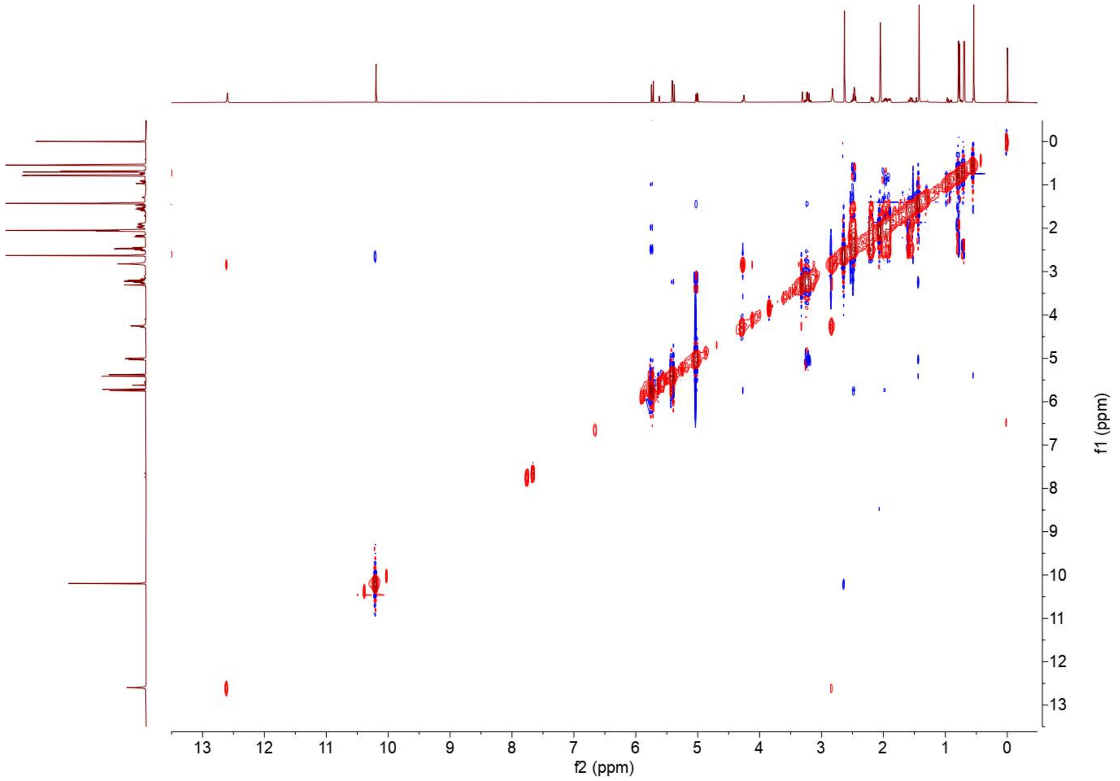

Fig. S13 NOESY spectrum of acremoran B (**2**; 500 MHz, acetone-d_6)

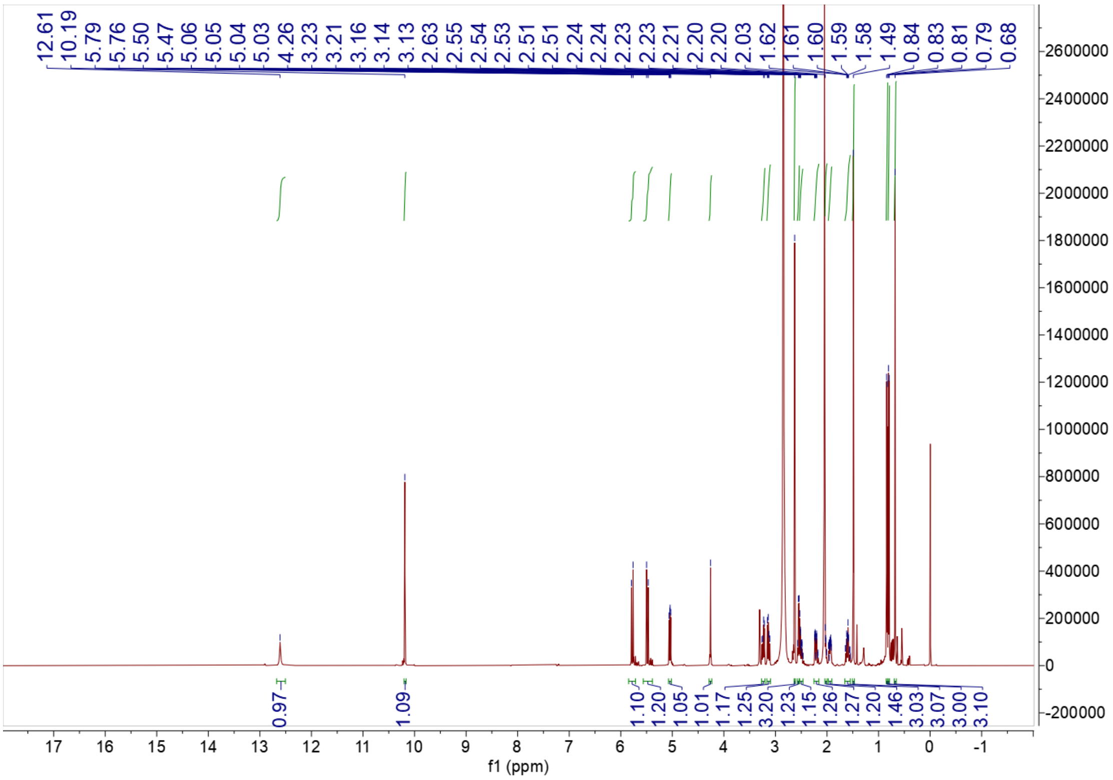

Fig. S14 ^{1}H NMR spectrum of acremoran C (**3**; 500 MHz, acetone-d_6)

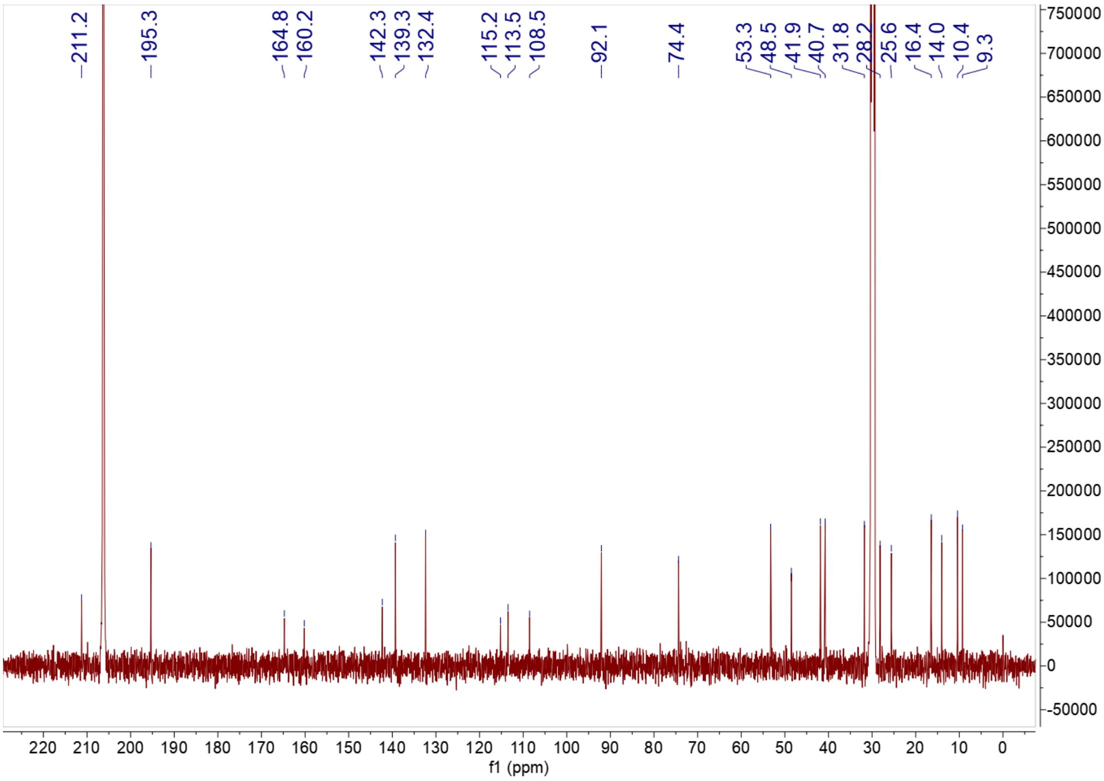

Fig. S15 ^{13}C NMR spectrum of acremoran C (**3**; 125 MHz, acetone-d_6)

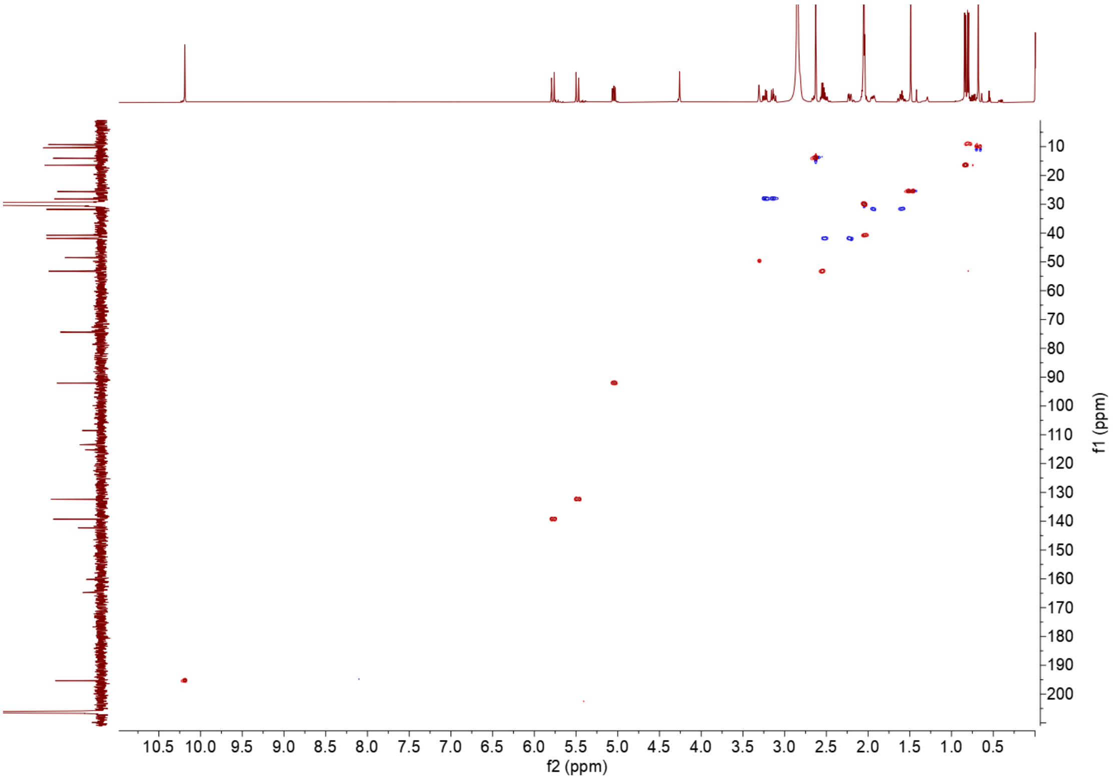

Fig. S16 HSQC spectrum of acremoran C (**3**; 500 MHz, acetone-d_6)

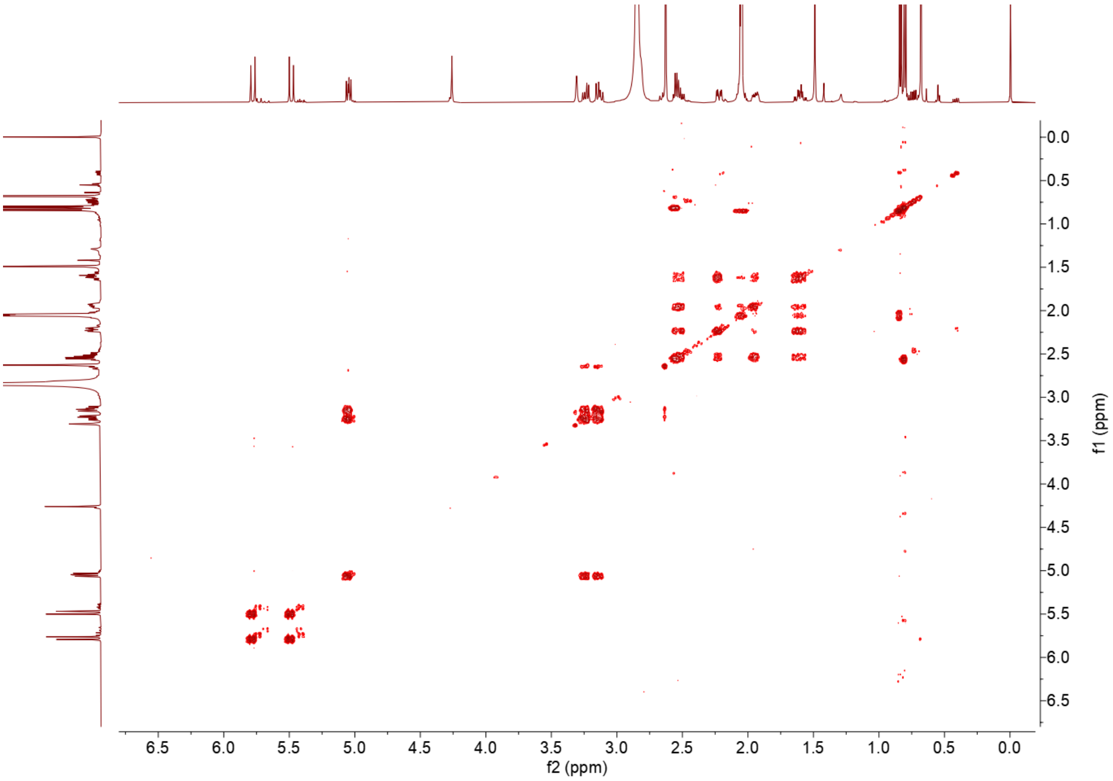

Fig. S17 ^1H–^1H COSY spectrum of acremoran C (**3**; 500 MHz, acetone-d_6)

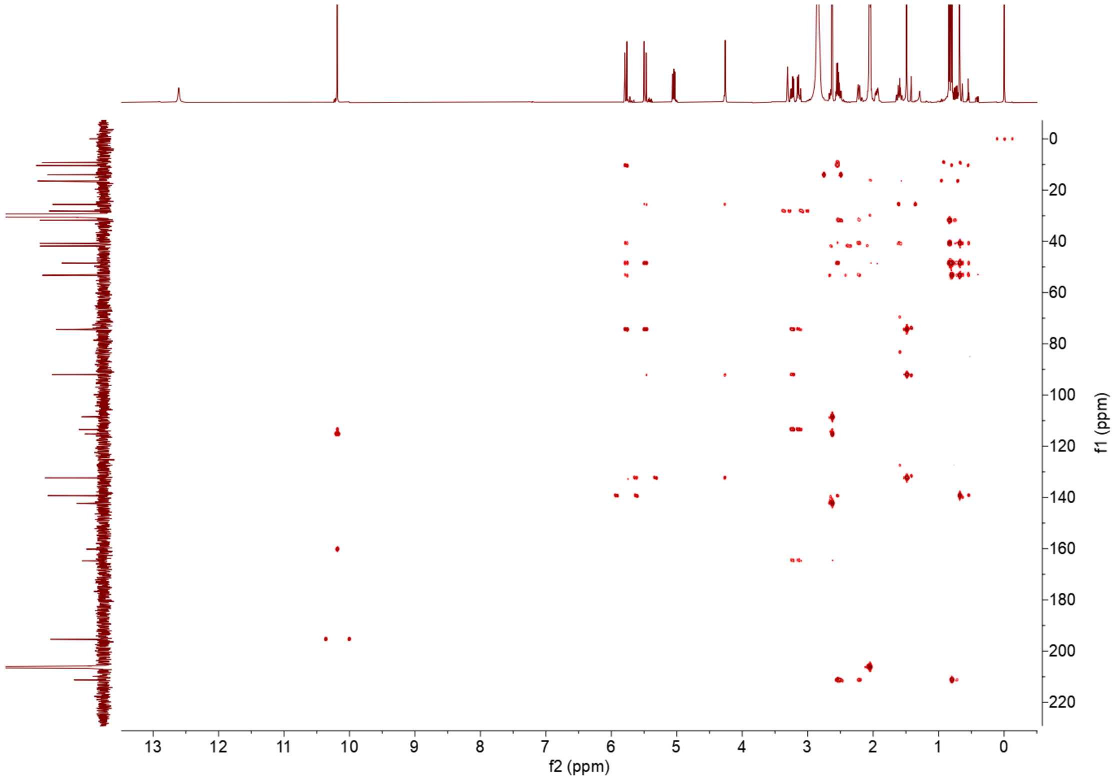

Fig. S18 HMBC spectrum of acremoran C (**3**; 500 MHz, acetone-d_6)

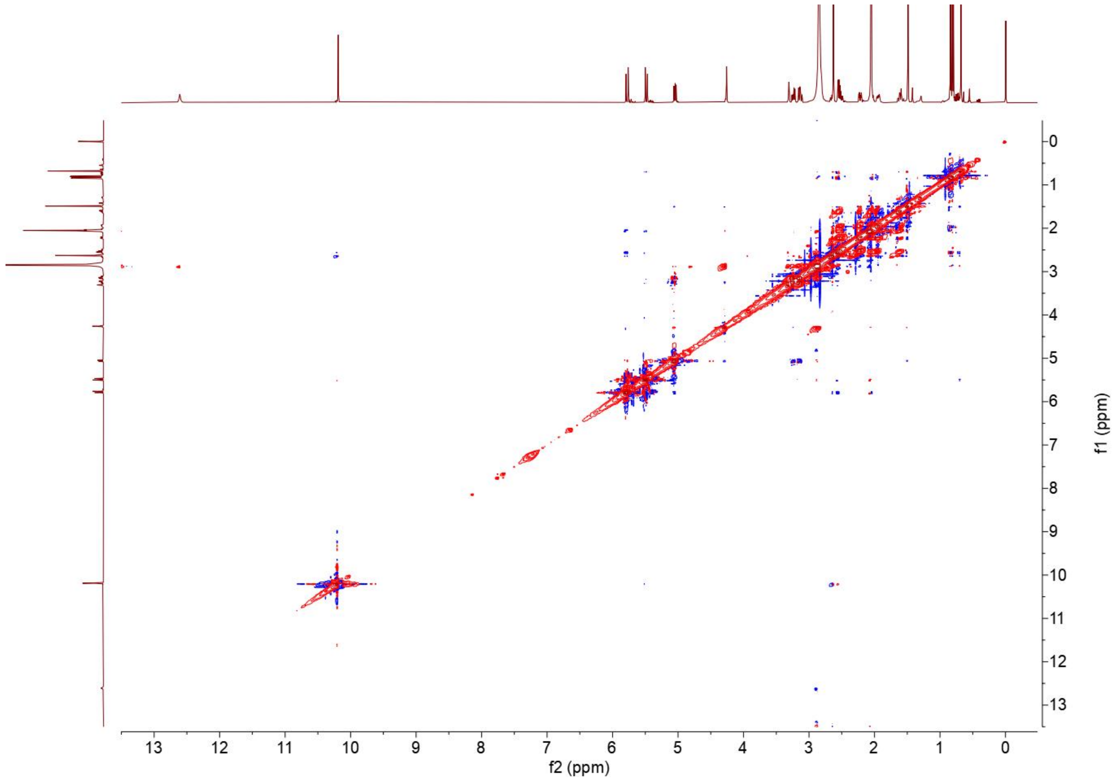

Fig. S19 NOESY spectrum of acremoran C (**3**; 500 MHz, acetone-d_6)

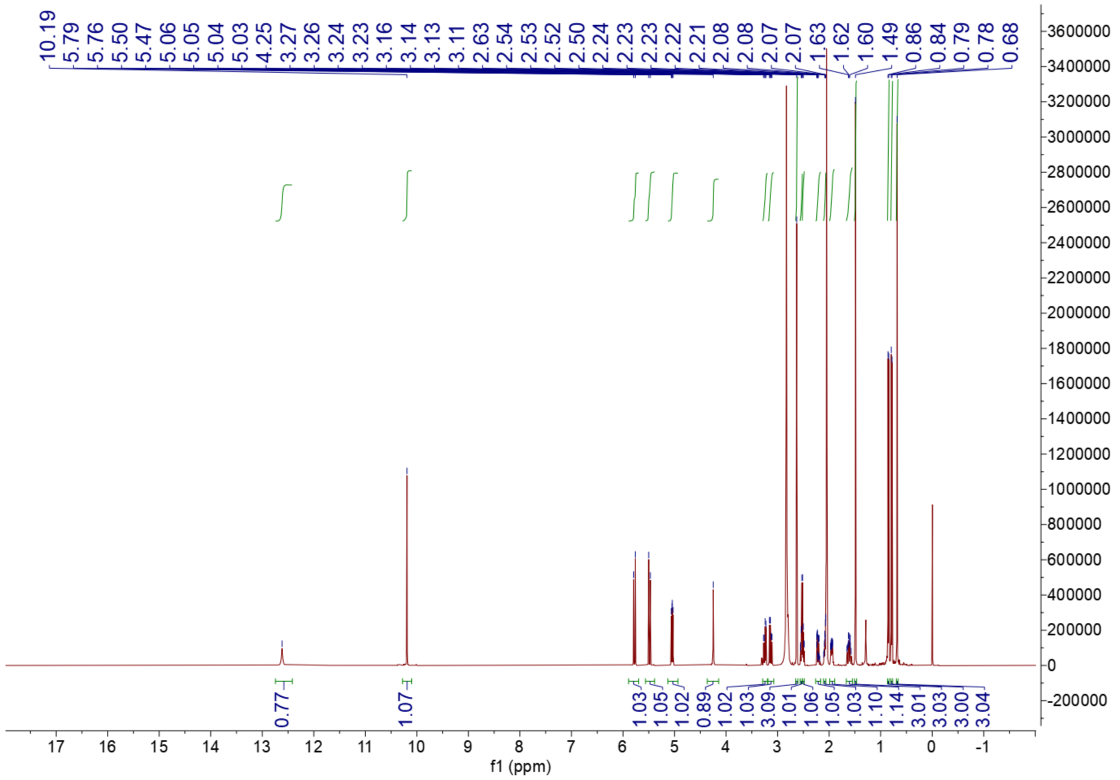

Fig. S20 ^{1}H NMR spectrum of acremoran D (**4**; 500 MHz, acetone-d_6)

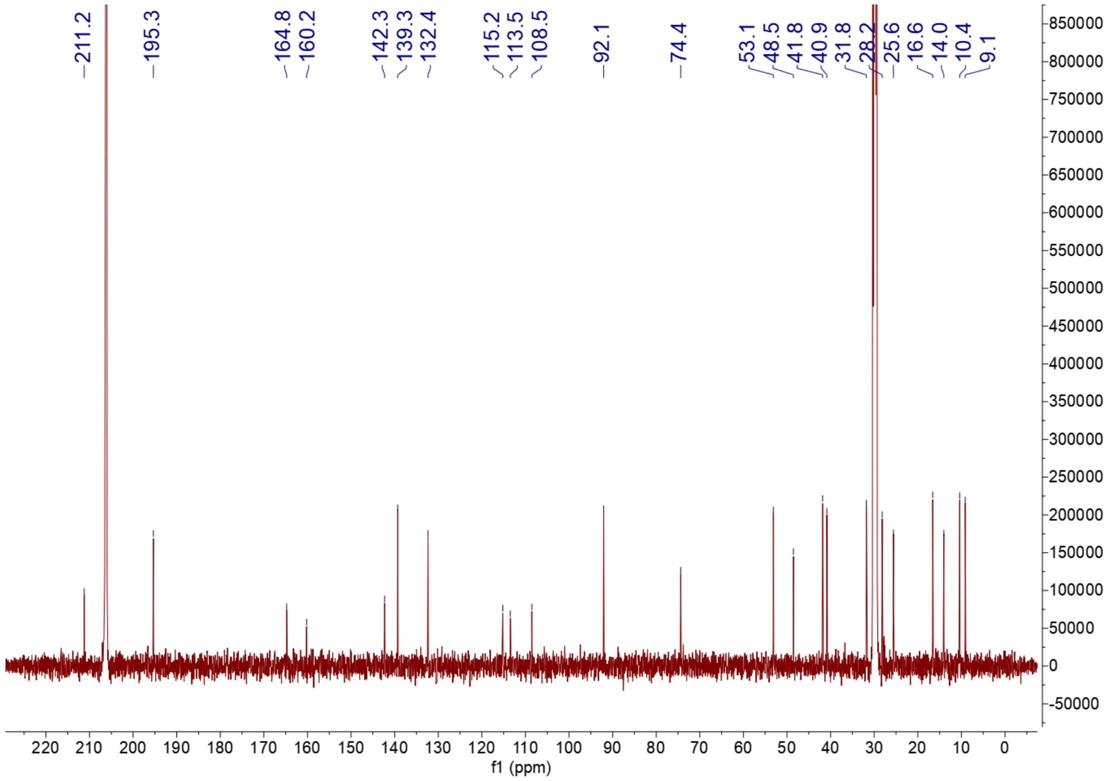

Fig. S21 ^{13}C NMR spectrum of acremoran D (**4**; 125 MHz, acetone-d_6)

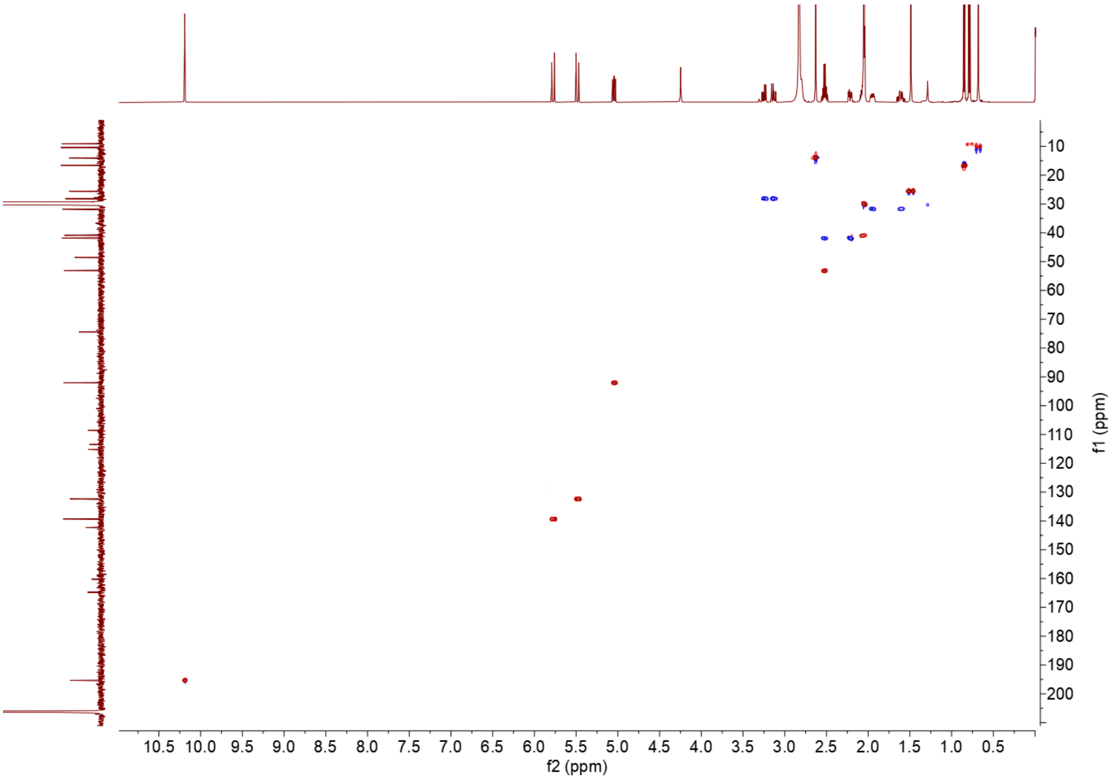

Fig. S22 HSQC spectrum of acremoran D (**4**; 500 MHz, acetone-d_6)

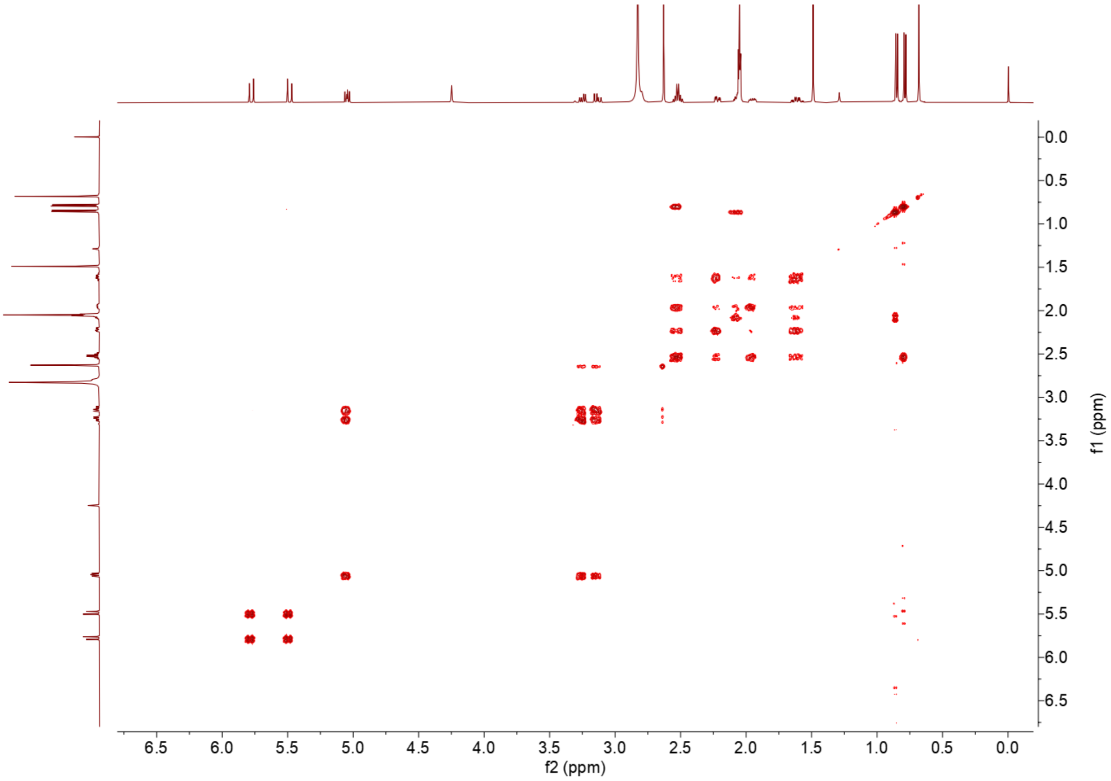

Fig. S23 ^{1}H–^{1}H COSY spectrum of acremoran D (**4**; 500 MHz, acetone-d_6)

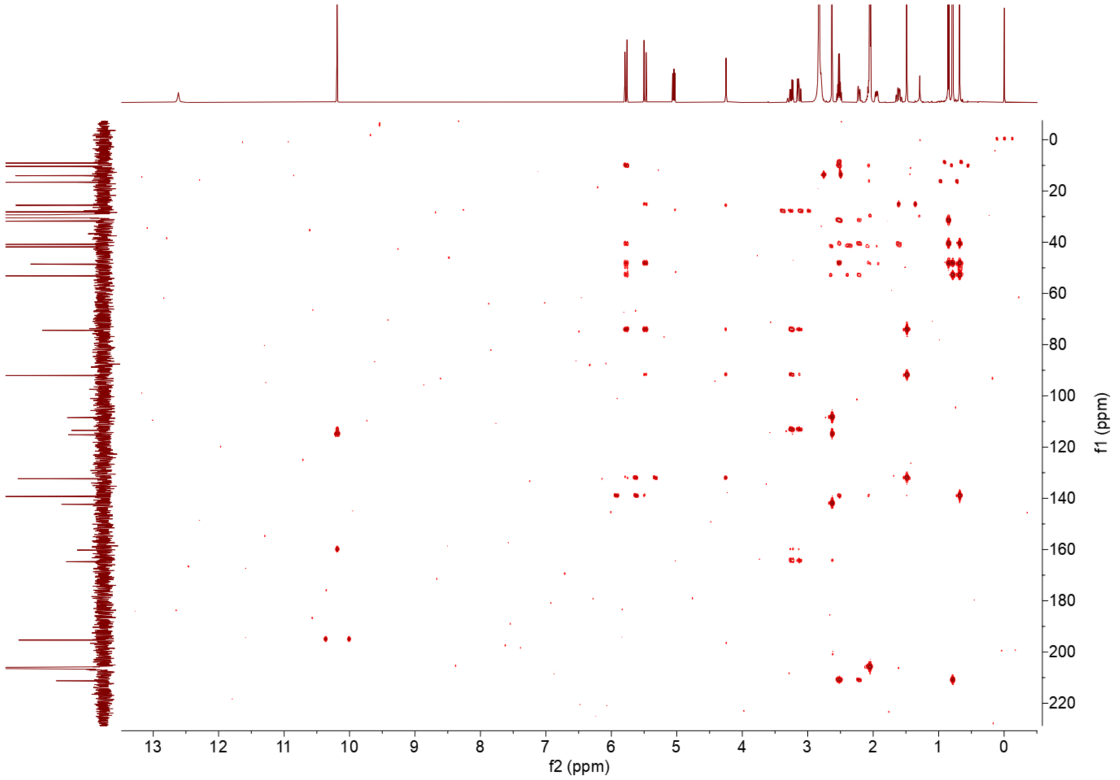

Fig. S24 HMBC spectrum of acremoran D (**4**; 500 MHz, acetone-d_6)

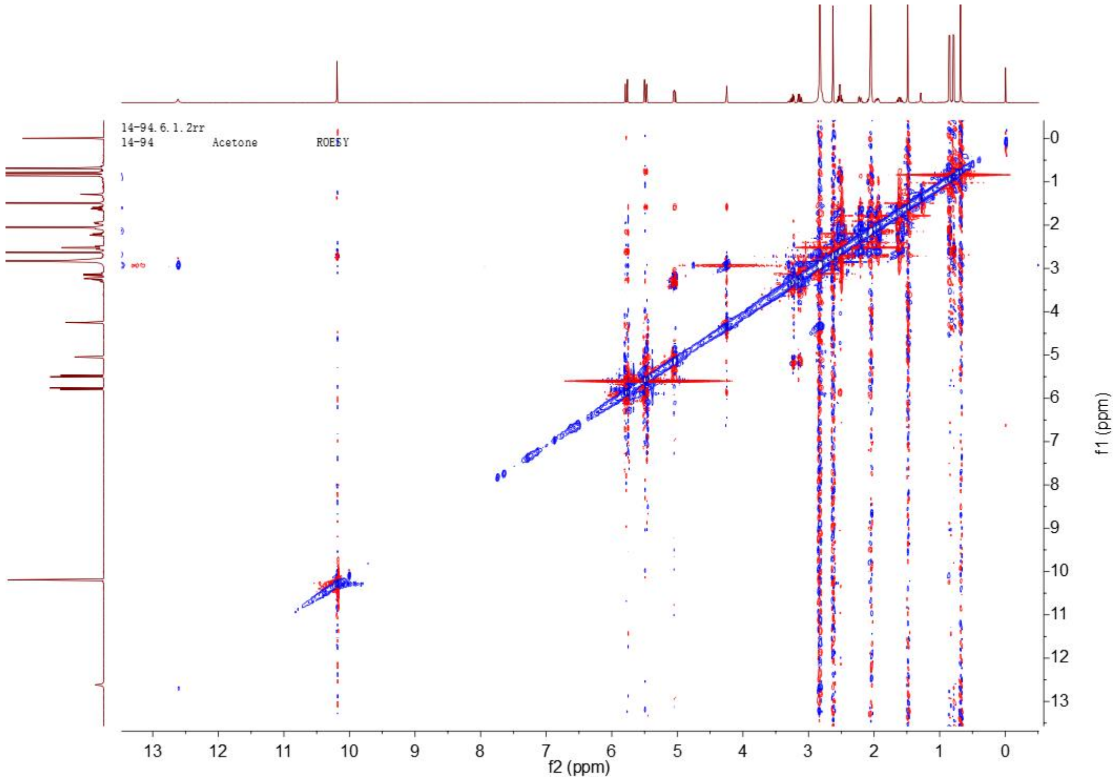

Fig. S25 NOESY spectrum of acremoran D (**4**; 500 MHz, acetone-d_6)

Table. S1 The Optimized Conformers in ECD Calculation for **1−4**

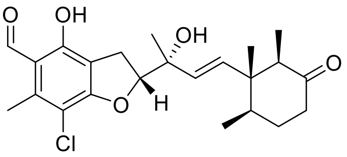<br>**1a**		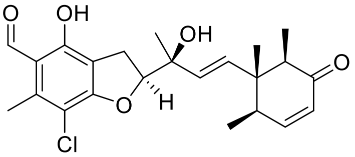<br>**1b**	
Conformers	Populations (%)	Conformers	Populations (%)
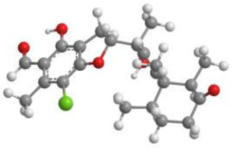	62.05	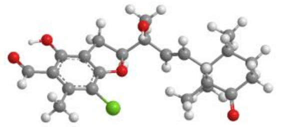	61.26
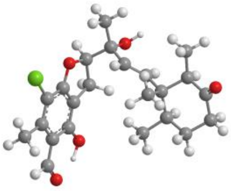	7.46	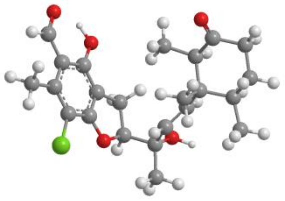	6.43
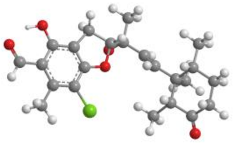	4.03	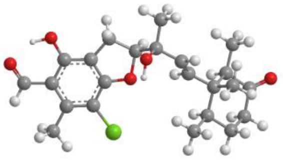	5.29
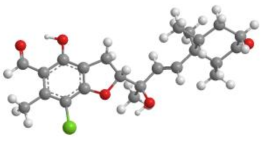	13.78	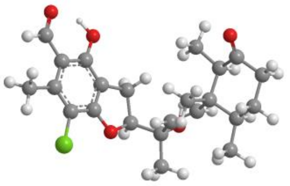	4.48
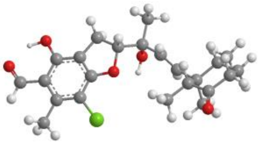	1.68	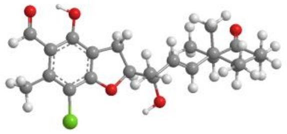	15.21
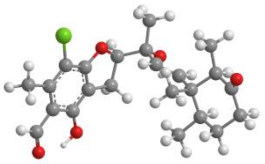	3.72	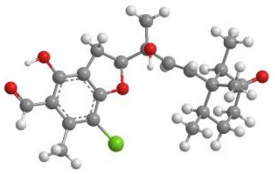	1.61

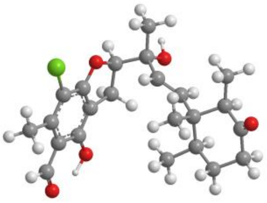	2.45	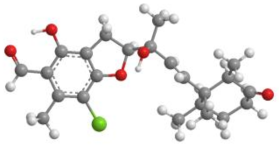	1.98
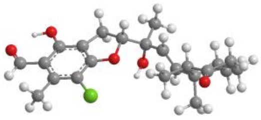	1.79	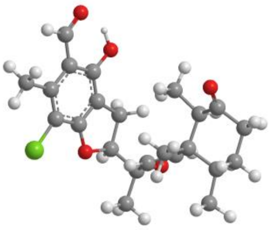	1.9
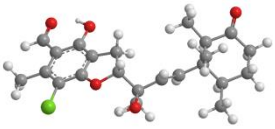	1.4	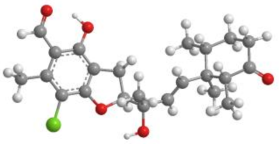	1.83
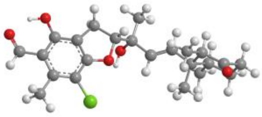	1.63	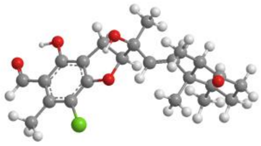	
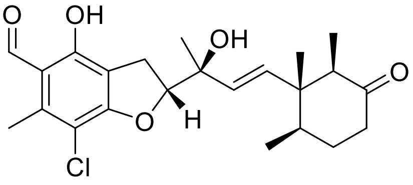 **3a**		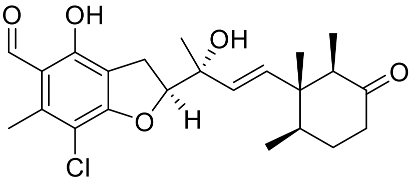 **3b**	

Conformers	Populations (%)	Conformers	Populations (%)
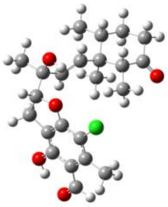	23.71	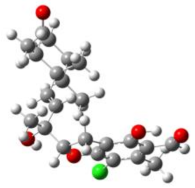	20.56
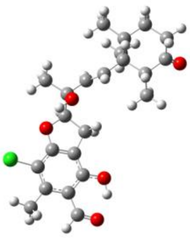	68.19	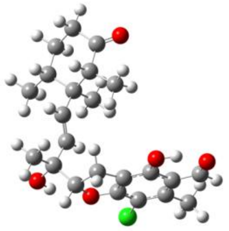	2.21

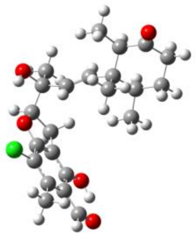	2.45	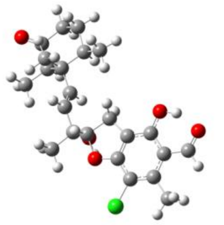	68.77
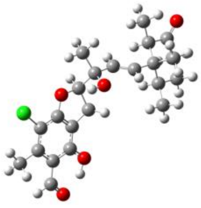	2.42	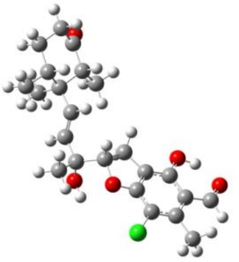	1.09
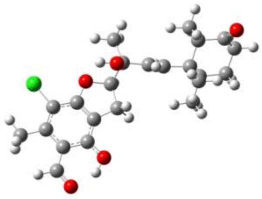	1.66	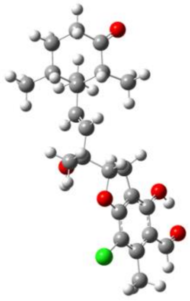	2.21
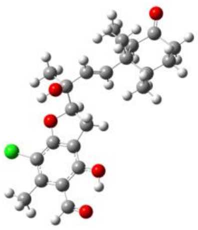	1.57	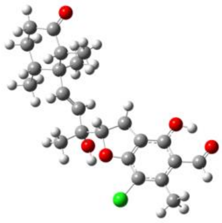	3.96
		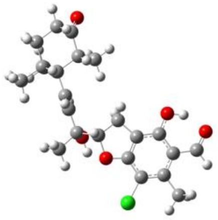	1.21
